# Disparities in Colorectal Cancer Incidence Trends Among Hispanics Living in Puerto Rico (2000–2021): A Comparison With Surveillance, Epidemiology, and End Results (SEER) Database

**DOI:** 10.1002/cam4.70851

**Published:** 2025-04-18

**Authors:** Luis D. Borrero‐Garcia, Marilyn Moró‐Carrión, Carlos R. Torres‐Cintrón, Hilmaris Centeno‐Girona, Victoria Perez, Taymaraliz Santos‐Colón, María González‐Pons

**Affiliations:** ^1^ Division of Clinical and Translational Cancer Research University of Puerto Rico Comprehensive Cancer Center San Juan Puerto Rico; ^2^ Puerto Rico Central Cancer Registry San Juan Puerto Rico; ^3^ School of Public Health University of Texas Health Science Center Houston Dallas Texas USA; ^4^ School of Public Health University of Puerto Rico Medical Sciences Campus San Juan Puerto Rico

**Keywords:** colorectal cancer, disparities, early‐onset colorectal cancer, Hispanic, racial and ethnic

## Abstract

**Background:**

Although the overall colorectal cancer (CRC) incidence has been steadily declining in the United States, a dramatic increase in the number of CRC cases among individuals younger than 50 years of age (early‐onset CRC) has been observed. CRC is the second and first leading cause of cancer death in the United States and among Hispanic men and women living in Puerto Rico (PRH), respectively. We report CRC incidence rates from 2000 to 2021 among PRH and compare them to data in the Surveillance, Epidemiology, and End Results Program (SEER).

**Methods:**

Data on colorectal adenocarcinomas diagnosed between January 1, 2000, and December 31, 2021, were obtained from the Puerto Rico Central Cancer Registry and SEER17, including race and ethnicity. Age‐standardized incidence rates were calculated using the direct method. The Joinpoint Regression Program calculated temporal trends on CRC incidence rates based on age‐adjusted Average Annual Percent Change (AAPC) estimates.

**Results:**

A total of 729,479 incident cases of CRC were analyzed. US Hispanics had the highest percentage of early‐onset CRC (EOCRC) cases (17.0%) among the racial and ethnic groups studied. PRH had the highest age‐standardized EOCRC incidence rate (12.18 per 100,000 persons) and the highest increase in EOCRC incidence temporal trends (AAPC = 2.68; 95% CI: 1.83 to 3.51).

**Conclusions:**

A significantly higher increase in EOCRC incidence was observed among Hispanic populations. Future studies should disaggregate Hispanic subpopulations by considering the country of ancestral origin, which will help identify specific risk factors and exposures and aid in developing tailored prevention and risk stratification strategies to reduce EOCRC incidence.

## 1 Introduction

Nearly 1.9 million colorectal cancer (CRC) cases are diagnosed each year globally [1, 2]. Although CRC is potentially preventable, it continues to be the second most diagnosed cancer in the United States and Puerto Rico [3]. Despite a decline in overall CRC incidence, the incidence of CRC among individuals younger than 50 years old has been rising consistently since the mid‐1990s [4, 5, 6]. Cases of colon or rectal cancer occurring in adults younger than 50 years old have largely been defined as early‐onset CRC (EOCRC) [4, 5, 6].

Approximately 20% of all CRC cases in the United States are diagnosed in individuals 54 years or younger [4]. Projections for EOCRC incidence between 2020 and 2030 indicate that these trends will continue, with increases of up to 124% for individuals between 20 and 34 years of age and up to 46% for individuals between 35 and 49 years of age [7]. The incidence of EOCRC has been reported to be rising more rapidly among Hispanic individuals compared to those in other racial and ethnic groups [8]. Current screening guidelines for average‐risk individuals begin at age 45 in the United States [9]. The Puerto Rico Department of Health recommended that average‐risk individuals begin routine CRC screening at age 40, becoming the territory with the lowest recommended CRC screening age in the United States [10]. However, a high percentage of individuals with EOCRC fall outside of these screening guidelines, leading to a higher number of diagnoses at advanced stages [11].

Almost 5.6 million Puerto Ricans live in the mainland United States, representing the second‐largest Hispanic group [12]. Puerto Rico is home to an additional 3.4 million people [12]; the majority (98.7%) identify as having Hispanic or Latino origin, regardless of race [13]. Although CRC is the leading cause of cancer‐related death in Hispanic individuals living in Puerto Rico (PRH) [3, 14], very little is known regarding EOCRC epidemiological trends in this subpopulation compared to individuals residing in the United States mainland, where CRC represents a malignancy with documented disparities among racial and ethnic groups [4, 15, 16, 17]. In this updated analysis, we describe incidence trends for CRC during 2000–2021 in Puerto Rico by age group, biological sex, and race and ethnicity using data from the Puerto Rico Central Cancer Registry, which is not included in the Surveillance, Epidemiology, and End Results Program (SEER), and compare trends to those from other racial and ethnic groups from the mainland United States using data available from SEER17.

## 2 Materials and Methods

### 2.1 Data Sources

CRC incidence data for PRH were provided by the Puerto Rico Central Cancer Registry (PRCCR) [14], which is currently not included in the SEER Program. Harmonization (coding, data gathering, and reporting) was performed in accordance with the North American Association of Central Cancer Registries (NAACCR) guidelines [18]. CRC incidence data, including stage at diagnosis, from racial and ethnic groups in the United States were obtained from SEER 17 [19]. For American Indians and Alaska Native data, SEER frequently only includes cases that are in a Purchased/Referred Care Delivery Area (PRCDA) to reduce racial misclassification.

Only primary cases with a diagnostic and histologic confirmation of colorectal adenocarcinomas from January 1, 2000, to December 31, 2021, were included (ICD‐O‐3 codes C18.0, C18.2–C18.9, C26.0, C19.9, and C20.9, which correspond to colon and rectal tumors). For our analyses, all cases were combined as CRC. EOCRC was defined as CRC diagnosed in an individual aged 20–49 years, while average‐onset CRC (AOCRC) was defined as CRC diagnosed in an individual aged ≥ 50 years. This secondary data study was conducted according to the guidelines of the Declaration of Helsinki and approved by the University of Puerto Rico Comprehensive Cancer Center Institutional Review Board (IRB # 2022‐10‐88). The informed consent does not apply to this study since the data came from a secondary database from the PRCCR, and there are minimal risks to subjects.

### 2.2 Demographic and Clinical Characteristics

Demographic and clinical characteristics were analyzed for the whole study period (2000–2021) by age percentiles (p25, p50, p75), age group (overall, 20‐49 years, and ≥ 50 years), sex (male vs. female), tumor location (right or left colon, colon‐NOS [not otherwise specified], or rectum), and cancer stage at diagnosis (localized, regional, distant, or unknown); and per reported racial and ethnic group (PRH, non‐Hispanic White [NHW], non‐Hispanic Black [NHB], non‐Hispanic American Indians/Alaska Natives [NHAI/AN], non‐Hispanic Asian or Pacific Islander [NHAPI], and United States Hispanic [USH]). USH includes individuals from diverse countries of origin, which may include Puerto Ricans who live in the mainland United States.

### 2.3 Age‐Standardized Rates

We calculated current (2017–2021) age‐standardized rates of CRC incidence (per 100,000 persons) with the direct method and the 2000 United States standard population [20, 21] using SEER*Stat v8.4.4. Data were analyzed by racial and ethnic group (PRH, NHW, NHB, NHAPI, NHAI/AN, and USH), sex (male vs. female), and age group (overall, 20–49 years, and ≥ 50 years).

### 2.4 Age‐Standardized Average Annual Percent Change

The National Cancer Institute's (NCI's) Joinpoint Regression Program (Version 5.2.0.0) was used to estimate age‐standardized average annual percent change (AAPC) from 2000 to 2019, quantify and visualize joinpoints, and perform parallelism and coincident tests (Supplementary table S1). Joinpoint applies permutation analysis to fit a sequence of connected straight lines on a logarithmic scale. The analysis uses a log‐linear model and the Grid Search Method to identify optimal joinpoints from the slope of the model. Data from 2020 and 2021 were excluded only in AAPC calculations to avoid bias because of lower CRC diagnosis and screening during the COVID‐19 pandemic [22, 23]. AAPCs were considered increasing or decreasing when considered statistically significant (two‐sided *p*‐value < 0.05). Data are presented as AAPC with 95% confidence intervals (95% CI) [24]. AAPC 95% confidence intervals were calculated using the empirical quantile method [25]. Data were analyzed by sex (male vs. female), age group (overall, 20–49 years, and ≥ 50 years), and cancer stage at diagnosis (localized, regional, or distant) per racial and ethnic group (PRH, NHW, NHB, NHAPI, NHAI/AN, and USH). The parallelism test and coincident test were used in a pairwise comparison to assess whether the trends of the two groups and rates of the two groups were similar throughout the study period (Table S1).

## 3 Results

### 3.1 CRC Incidence by Racial and Ethnic Group

Demographic and clinical characteristics for a total of 734,312 incident CRC cases by race and ethnic group during the 21‐year period are shown in Table 1. Overall, USH showed the highest percentage (17.0%) of incident cases in individuals younger than 50 years of age, and NHW had the highest percentage (91.7%) of cases in individuals 50 years or older. PRH and NHB had the highest rates of male and female incident cases, respectively. NHB also had the highest percentage of tumors located in the right colon (44.8%) and distant stage at diagnosis (24.7%).

**TABLE 1:** Demographic and clinical characteristics for incident CRC among individuals ≥ 20 years of age by racial and ethnic group; 2000–2021.

Characteristic	PRH	NHW	NHB	NHAPI	NHAI/AN	USH
	*n* = 32,181 (%)	*n* = 489,948 (%)	*n* = 74,753 (%)	*n* = 56,700 (%)	*n* = 4833 (%)	*n* = 75,897 (%)
Age percentiles (years)						
p25	59	60	55	56	54	53
p50	68	70	65	66	64	63
p75	77	79	74	76	73	74
Age group						
20–49	2853 (8.9)	40,556 (8.3)	9200 (12.3)	7285 (12.8)	741 (15.3)	12,878 (17.0)
50+	29,328 (91.1)	449,392 (91.7)	65,553 (87.7)	49,415 (87.2)	4092 (84.7)	63,019 (83.0)
Sex						
Male	17,817 (55.4)	257,908 (52.6)	37,719 (50.5)	29,997 (52.9)	2481 (51.3)	41,522 (54.7)
Female	14,364 (44.6)	232,040 (47.4)	37,034 (49.5)	26,703 (47.1)	2352 (48.7)	34,375 (45.3)
Location						
Right colon	11,654 (36.2)	206,915 (42.2)	33,505 (44.8)	17,325 (30.6)	1766 (36.5)	27,378 (36.1)
Left colon	10,070 (31.3)	131,448 (26.8)	21,601 (28.9)	19,660 (34.7)	1406 (29.1)	22,101 (29.1)
Colon, NOS	1450 (4.5)	12,902 (2.6)	2814 (3.8)	1170 (2.1)	134 (2.8)	2279 (3.0)
Rectum	9007 (28.0)	138,683 (28.3)	16,833 (22.5)	18,545 (32.7)	1527 (31.6)	24,139 (31.8)
Stage at diagnosis						
Localized	12,580 (39.1)	196,150 (40.0)	26,472 (35.4)	20,597 (36.3)	1707 (35.3)	26,788 (35.3)
Regional	12,958 (40.3)	185,741 (37.9)	26,935 (36.0)	22,927 (40.4)	1897 (39.3)	29,866 (39.4)
Distant	4318 (13.4)	91,379 (18.7)	18,455 (24.7)	11,001 (19.4)	1089 (22.5)	16,294 (21.5)
Unknown	2325 (7.2)	16,659 (3.4)	2889 (3.9)	2170 (3.8)	140 (2.9)	2941 (3.9)

Abbreviations: NHAI/AN = non‐Hispanic American Indians/Alaska Natives, NHAPI = non‐Hispanic Asian or Pacific Islanders, NHB = non‐Hispanic‐Blacks, NHW = non‐Hispanic Whites, NOS = not otherwise specified, PRH = Hispanics living in Puerto Rico, USH = United States Hispanics.

Among the racial and ethnic groups studied, NHAI/AN had the highest overall (51.89) and female (47.21) age‐standardized CRC incidence rates, including individuals diagnosed at 20–49 (14.79) and ≥ 50 years old (98.45) (Table 2). NHB showed the highest age‐standardized CRC incidence rates among men overall and those aged ≥ 50 years. NHAI/AN and PRH had the highest age‐standardized incidence rates of EOCRC overall (15.32 and 12.18, respectively) and among women with EOCRC (14.79 and 11.42, respectively) compared to the other racial and ethnic groups studied. The highest age‐standardized CRC incidence rates among men 20–49 years old were observed in NHAI/AN and NHW.

**TABLE 2:** Age‐standardized incidence rates^a^ (per 100,000 persons) for CRC by racial and ethnic group; 2017–2021.

	PRH	NHW	NHB	NHAPI	NHAI/AN	USH	Overall US
Overall	42.66	43.95	49.30	36.33	51.89	38.34	43.05
Age group							
20–49 yo	12.18	11.80	11.71	9.32	15.32	9.57	11.01
> 50 yo	90.84	94.78	108.71	79.03	109.70	83.82	93.71
Sex							
Male	52.82	50.62	58.45	43.14	57.11	45.21	49.82
Males by age							
20–49 yo	13.02	13.17	12.55	9.88	15.85	9.73	11.92
> 50 yo	115.71	109.82	131.01	95.71	122.32	101.28	109.70
Female	34.46	37.93	42.56	30.81	47.21	32.72	37.20
Females by age							
20–49 yo	11.42	10.35	10.94	8.81	14.79	9.40	10.07
> 50 yo	70.88	81.51	92.52	65.57	98.45	69.58	80.08

Abbreviations: NHAI/AN = non‐Hispanic American Indians/Alaska Natives, NHAPI = non‐Hispanic Asian or Pacific Islanders, NHB = non‐Hispanic Blacks, NHW = non‐Hispanic Whites, Overall US = all United States, including all racial and ethnic groups, PRH = Hispanics living in Puerto Rico, USH = United States Hispanics, yo = years old.

^a^ Age‐standardized incidence rates using the US 2000 standard population.

### 3.2 Age‐Adjusted AAPCs by Racial and Ethnic Group

The temporal trends in overall CRC incidence for all racial and ethnic groups are shown in Table 3. Except for NHAI/AN and PRH, significantly decreasing overall CRC incidence trends were observed in all racial and ethnic groups during the study period. NHAI/AN had a significant increase in cases diagnosed at distant stages (AAPC = 2.21; CI: 1.14, 3.28). PRH had a significantly smaller decline in CRC incidence among women (AAPC = −0.89; CI: −01.43, −0.47) and a marked increase in cases diagnosed at distant stages (AAPC = 1.76; CI: 0.67, 2.86) compared to the other racial and ethnic groups studied, except for NHAI/AN.

**TABLE 3:** Age‐adjusted Average Annual Percent Change (AAPC) in CRC incidence by racial and ethnic group; 2000–2021.^a^

	PRH AAPC (95% CI)	NHW AAPC (95% CI)	NHB AAPC (95% CI)	NHAPI AAPC (95% CI)	NHAI/AN AAPC (95% CI)	USH AAPC (95% CI)	Overall US AAPC (95% CI)
Overall	−0.36 (−0.74 to 0.01)	−2.42* (−2.58 to −2.29)	−2.32* (−2.51 to −2.18)	−2.58* (−2.82 to −2.34)	0.71 (−0.25 to 1.45)	−1.56* (−1.77 to −1.36)	−2.48* (−2.59 to −2.34)
Sex							
Male	0.07 (−0.50 to 0.37)	−2.72* (−2.93 to −2.52)	−2.23* (−2.57 to −1.99)	−2.54* (−2.79 to −2.28)	0.47 (−0.81 to 1.72)	−1.73* (−1.93 to −1.47)	−2.53* (−2.72 to −2.39)
Female	−0.89* (−1.43 to −0.47)	−2.46* (−2.65 to −2.32)	−2.55* (−2.89 to −2.23)	−2.69* (−3.02 to −2.38)	0.26 (−0.25 to 0.77)	−1.53* (−1.74 to −1.31)	−2.44* (−2.54 to −2.32)
Stage at diagnosis							
Localized	0.53 (−0.21 to 1.25)	−2.99* (−3.21 to −2.81)	−2.64* (−3.00 to −2.23)	−2.58* (−2.95 to −2.27)	0.04 (−1.10 to 1.19)	−2.38* (−3.25 to −1.57)	−2.95* (−3.13 to −2.77)
Regional	−0.48 (−1.33 to 0.29)	−2.46* (−2.69 to −2.23)	−3.11* (−3.46 to −2.77)	−3.06* (−3.73 to −2.39)	0.47 (−0.05 to 1.12)	−1.77* (−2.31 to −1.25)	−2.52* (−2.81 to −2.24)
Distant	1.76* (0.67 to 2.86)	−0.84* (−1.10 to −0.62)	−1.53* (−1.89 to −1.18)	−1.52* (−2.03 to −1.02)	2.21* (1.14 to 3.28)	−0.56 (−1.13 to 0.004)	−0.78* (−0.99 to −0.61)

Abbreviations: NHAI/AN = non‐Hispanic American Indians/Alaska Natives, NHAPI = non‐Hispanic Asian or Pacific Islanders, NHB = non‐Hispanic Blacks, NHW = non‐Hispanic Whites, Overall US = all United States, including all racial and ethnic groups, PRH = Hispanics living in Puerto Rico, USH = United States Hispanics.

^a^ Years 2020 and 2021 were excluded from data analysis as incidence data from these years are not representative of actual CRC incidence due to the COVID‐19 pandemic.

* Two‐sided *p* < 0.05.

**FIGURE 1:**
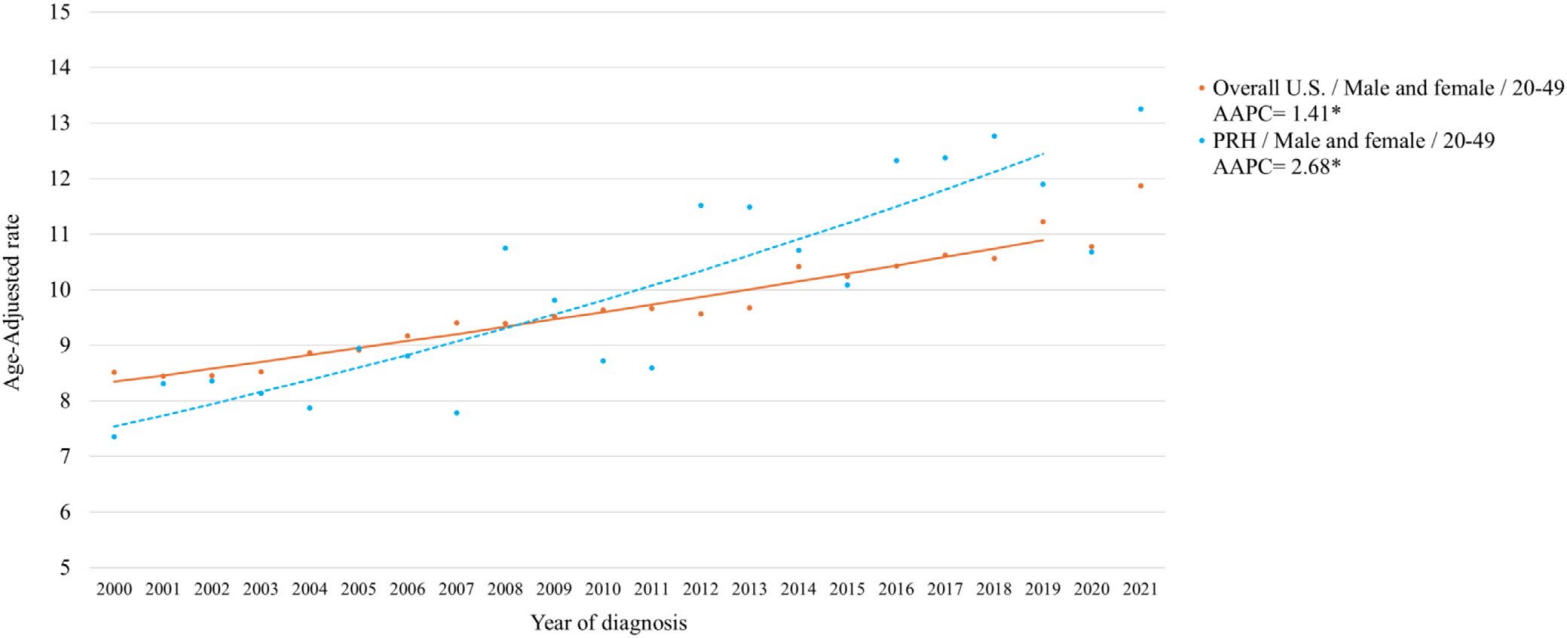
Age‐adjusted Average Annual Percent Change (AAPC) for EOCRC over 2000–2019 for PRH and the United States (US) overall. PRH and overall US EOCRC incidence trends are highlighted in blue and orange, respectively. *Denotes significant change in temporal trend.

During the study period, PRH showed significantly higher marked increases in EOCRC temporal trends regardless of sex and stage at diagnosis compared to the other racial and ethnic groups, apart from NHAI/AN (Table 4). Notably, the increasing temporal trend in EOCRC was higher in the PRH when compared with the overall United States (US) trend (Figure 1). Comparable AAPCs were observed between PRH and USH among females with EOCRC (AAPC = 2.41; CI: 1.43, 3.41 and AAPC = 2.48; CI: 1.81, 3.09, respectively) and among regional EOCRC (AAPC = 2.08; CI: 0.21, 3.99 and AAPC = 2.08; CI: 1.22, 2.92, respectively). The highest AAPCs among those aged 20–49 were observed in NHAI/AN; however, significantly increasing trends were only observed for overall EOCRC, females with EOCRC, and tumors diagnosed in localized and distant stages.

**TABLE 4:** Age‐adjusted Average Annual Percent Change (AAPC) in CRC incidence among individuals aged 20–49 years by racial and ethnic group; 2000–2021.^a^

	PRH AAPC (95% CI)	NHW AAPC (95% CI)	NHB AAPC (95% CI)	NHAPI AAPC (95% CI)	NHAI/AN AAPC (95% CI)	USH AAPC (95% CI)	Overall US AAPC (95% CI)
Overall	2.68* (1.83 to 3.51)	1.90* (1.64 to 2.16)	0.41 (−0.06 to 0.89)	0.08 (−0.42 to 0.56)	5.08* (2.65 to 7.06)	2.21* (1.60 to 2.68)	1.41* (1.26 to 1.56)
Sex							
Male	2.99* (1.64 to 4.34)	2.00* (1.68 to 2.32)	0.25 (−0.39 to 0.87)	0.09 (−1.03 to 1.19)	2.48 (−0.50 to 5.66)	1.88* (1.37 to 2.38)	1.42* (1.15 to 1.69)
Female	2.41* (1.43 to 3.41)	1.52* (1.06 to 1.94)	0.61 (−0.19 to 1.38)	0.06 (−0.75 to 0.88)	3.95* (0.70 to 7.25)	2.48* (1.81 to 3.09)	1.39* (1.11 to 1.66)
Stage at diagnosis							
Localized	3.66* (1.74 to 5.59)	0.44 (−0.24 to 1.11)	0.02 (−1.05 to 1.08)	−1.62* (−2.81 to −0.43)	5.46* (1.08 to 10.01)	0.58 (−0.28 to 1.43)	0.03 (−0.52 to 0.58)
Regional	2.08* (0.21 to 3.99)	2.01* (1.64 to 2.39)	0.31 (−0.32 to 0.95)	0.62 (−0.39 to 1.45)	1.38 (−1.58 to 4.36)	2.08* (1.22 to 2.92)	1.48* (1.13 to 1.81)
Distant	4.77* (3.22 to 6.32)	3.87* (3.18 to 4.41)	1.02* (0.19 to 1.82)	1.18* (0.25 to 2.11)	8.46* (1.05 to 13.32)	3.37* (2.29 to 4.43)	2.88* (2.69 to 3.06)

Abbreviations: NHAI/AN = non‐Hispanic American Indians/Alaska Natives, NHAPI = non‐Hispanic Asian or Pacific Islanders, NHB = non‐Hispanic Blacks, NHW = non‐Hispanic Whites, Overall US = all United States, including all racial and ethnic groups, PRH = Hispanics living in Puerto Rico, USH = United States Hispanics.

^a^ Years 2020 and 2021 were excluded from data analysis as incidence data from these years are not representative of actual CRC incidence due to the COVID‐19 pandemic.

* Two‐sided *p* < 0.05.

**FIGURE 2:**
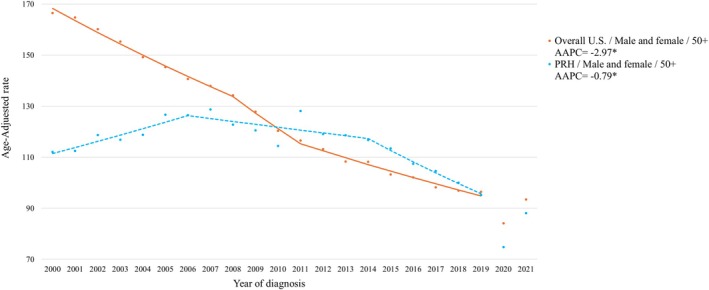
Age‐adjusted Average Annual Percent Change (AAPC) for AOCRC over 2000–2019 for PRH and the United States (US) overall. PRH and overall US AOCRC incidence trends are highlighted in blue and orange, respectively. *Denotes significant change in temporal trend.

Analysis of AAPCs for those aged ≥ 50 years showed overall declining trends in all the racial and ethnic groups studied, where NHW exhibited the largest decreasing trend in overall AOCRC (Table 5). In contrast to the overall US population, which demonstrated an earlier decline in incidence, PRH did not show a decreasing temporal trend in AOCRC until 2006 (see Figure 2). The most minor declining temporal trends were observed among NHAI/AN and PRH. An increasing trend in cases diagnosed at distant stages was observed among NHAI/AN.

**TABLE 5:** Age‐adjusted Average Annual Percent Change (AAPC) in CRC incidence among individuals aged 50+ years by racial and ethnic group; 2000–2021.^a^

	PRH AAPC (95% CI)	NHW AAPC (95% CI)	NHB AAPC (95% CI)	NHAPI AAPC (95% CI)	NHAI/AN AAPC (95% CI)	USH AAPC (95% CI)	Overall US AAPC (95% CI)
Overall	−0.79* (−1.13 to—0.47)	−2.96* (−3.11 to −2.85)	−2.64* (−2.83 to −2.49)	−2.95* (−3.22 to −2.67)	−0.09 (−0.50 to 0.32)	−2.01* (−2.24 to −1.79)	−2.97* (−3.08 to −2.83)
Sex							
Male	−0.42 (−0.87 to 0.03)	−3.23* (−3.43 to −2.99)	−2.50* (−2.86 to −2.23)	−2.86* (−3.11 to −2.61)	0.10 (−0.95 to 1.13)	−2.11* (−2.40 to −1.79)	−2.99* (−3.19 to −2.84)
Female	−1.59* (−2.31 to—1.03)	−2.99* (−3.19 to −2.84)	−2.95* (−3.21 to −2.71)	−3.05* (−3.70 to −2.47)	−0.21 (−0.80 to 0.38)	−2.02* (−2.28 to −1.77)	−2.93* (−3.02 to −2.83)
Stage at diagnosis							
Localized	0.22 (−0.56 to 0.93)	−3.32* (−3.52 to −3.17)	−2.99* (−3.36 to −2.61)	−2.71* (−3.15 to −2.34)	−0.50 (−1.81 to 0.81)	−2.61* (−3.37 to −1.89)	−3.24* (−3.46 to −3.07)
Regional	−0.93* (−1.79 to—0.12)	−3.10* (−3.33 to −2.86)	−3.08* (−3.39 to −2.72)	−3.66* (−4.26 to −3.23)	−0.83* (−1.56 to −0.11)	−2.26* (−2.73 to −1.79)	−3.09* (−3.36 to −2.80)
Distant	1.12 (−1.01 to 3.48)	−1.62* (−1.86 to −1.41)	−1.95* (−2.46 to −1.44)	−2.05* (−2.58 to −1.52)	1.74* (0.21 to 3.29)	−1.21* (−1.76 to −0.67)	−1.56* (−1.89 to −1.39)

Abbreviations: NHAI/AN = non‐Hispanic American Indians/Alaska Natives, NHAPI = non‐Hispanic Asian or Pacific Islanders, NHB = non‐Hispanic Blacks, NHW = non‐Hispanic Whites, Overall US = all United States, including all racial and ethnic groups, PRH = Hispanics living in Puerto Rico, USH = United States Hispanics.

^a^ Years 2020 and 2021 were excluded from data analysis as incidence data from these years are not representative of actual CRC incidence due to the COVID‐19 pandemic.

* Two‐sided *p* < 0.05.

## 4 Discussion

CRC is one of the leading causes of cancer death in the United States and Puerto Rico, with persistent disparities in incidence and survival among racial and ethnic groups [15, 26]. Puerto Ricans are the second largest subpopulation of Hispanics in the United States, accounting for approximately 9% of all Hispanics [12, 27]. Notably, differences in CRC incidence, including EOCRC, have been reported among Hispanics according to their country of origin [28, 29]. Despite the observed disparities, there is a scarcity of updated information on CRC epidemiology for individuals living on the island, which is crucial for developing tailored CRC control programs. This lack of data on CRC epidemiology in Puerto Rico has prompted this analysis, which compares the updated incidence trends of PRH to other racial and ethnic groups in the United States available through the SEER 17 database over a 22‐year period.

### 4.1 Overall and Average‐Onset CRC

When comparing the demographic and clinical characteristics of incident AOCRC according to race and ethnicity during 2000–2021, as previously reported, a higher number of CRC cases were diagnosed in men in all the racial and ethnic groups studied [30, 31]. The highest percentage of CRC diagnosed among men was observed in PRH, likely reflecting cultural factors influencing health‐seeking behaviors [32, 33]. NHB had the highest percentage of right colon and distant‐stage tumors, consistent with previous reports [34, 35, 36, 37].

The highest overall age‐standardized CRC incidence rates during 2017–2021 were observed among NHAI/AN, followed by NHB and NHW, consistent with age‐adjusted CRC incidence rates reported for 2015–2019 [30]. Although PRH had comparable overall incidence rates to the United States mainland population, AOCRC rates were slightly lower than the United States population but higher than USH. This difference calls for additional research to identify the factors contributing to the increased rate compared to USH.

When analyzing incidence trends from 2000 to 2019, decreasing trends were observed for overall and AOCRC incidence for all racial and ethnic groups, similar to previous studies [4, 30]; they do support a disparate CRC among PRH. The less marked decreases in AAPCs in all the categories studied could be a result of lower adherence to routine screening among the other potential unmodifiable and modifiable factors discussed in detail below. When considering the overall incidence trends, the increasing trend among males and localized tumors in PRH could also be attributed to the increasing trends in EOCRC.

### 4.2 Early‐Onset CRC Trends

Similar to previous reports, USH had the highest percentage of EOCRC compared to racial and ethnic groups studied [38, 39]. Between 2017 and 2021, NHAI/AN populations had the highest overall age‐standardized EOCRC incidence rates, followed by PRH. While increasing EOCRC incidence rates among NHAI/AN have been previously reported [40], the underlying causes remain poorly understood. It is possible that some of the non‐modifiable and modifiable factors discussed below, which we postulate contribute to the disproportionate EOCRC burden among PRH, may also play a role in driving the concerning increase in EOCRC rates among NHAI/AN. In our analyses, while NHAI/AN populations show significant increases in EOCRC incidence, the unique disparities seen in PRH highlight the role of specific environmental and genetic factors. Interestingly, PRH and USH showed comparable EOCRC AAPCs among women and those diagnosed with regional disease, possibly reflecting the fact that Hispanic women are more likely to undergo CRC screening [41]. However, EOCRC incidence trends among men were the highest among PRH, which could be partly attributed to men having less health‐seeking behaviors when presenting symptoms [42]. The significantly higher increasing EOCRC incidence trends observed for tumors diagnosed at both localized and distant stages compared to other groups, including USH, could stem from differences in the combination of modifiable and non‐modifiable factors. While PRH's lower recommended CRC screening age may help address these trends, adherence to this guideline remains uncertain and requires further evaluation. To better understand these trends, it is important to explore the potential genetic, environmental, and behavioral factors that may contribute to the observed disparities among PRH.

### 4.3 Key Contributors to CRC Disparities in PRH

Puerto Rico, a United States island territory located nearly 1000 miles from the mainland, may expose its residents to unique environmental and lifestyle factors contributing to the observed higher CRC incidence rates. A study analyzing 218 CRC tumors from PRH identified distinct actionable mutation profiles [43], supporting the role of unique exposures and lifestyle factors in shaping tumor mutational profiles. Approximately 75% of all CRCs are sporadic, non‐familial cases, whereas 5%–10% are caused by inherited pathogenic mutations in high‐penetrance genes [44, 45]. Therefore, we believe that factors increasing the incidence of sporadic CRC are primarily responsible for the disparities observed among PRH.

When considering the factors contributing to sporadic CRC incidence disparities among PRH, in general, risk factors can be classified as unmodifiable (e.g., genetic susceptibility) or modifiable (e.g., socioeconomic, behavioral, and environmental factors) [46, 47, 48, 49]. Puerto Ricans are an admixed population of varying degrees of three ancestral populations: European, African, and Amerindian (Taínos). Studies evaluating the association between ancestry and CRC clinicopathological characteristics in 831 PRH individuals found that genetic similarity to Africans and Amerindians was associated with rectal tumors and EOCRC, respectively [50, 51].

Modifiable socioeconomic, behavioral, and environmental factors, such as low socioeconomic status, inadequate health access, limited CRC screening awareness, poor diet, high obesity rates, and low physical activity levels, may contribute to the disparate CRC incidence trends among PRH compared to other groups [31, 52, 53, 54]. CRC incidence is disproportionately higher in populations with lower socioeconomic status due to delayed diagnosis, limited access to care, and reduced adherence to treatment [55, 56]. These challenges highlight the need for additional research that addresses how social determinants influence the observed CRC incidence trends [53, 54]. In Puerto Rico, CRC screening adherence rates were 14.1% lower in 2014, 10% lower in 2016, and 13.7% lower in 2018 compared to those living in the United States (50 states and D.C.), potentially delaying diagnoses and exacerbating the observed disparities [57]. High obesity rates, insufficient physical activity, and unique dietary habits among PRH compared to other populations may also contribute to these disparities, warranting further analysis [57, 58]. Additionally, differences in the frequencies of actionable mutations, MSI, and CIMP status of colorectal tumors from PRH, compared to other populations in the United States, support the notion that distinct genetic and environmental factors may influence tumor characteristics in this population and may contribute to the observed disparities [43, 51]. Further research into the interaction of the abovementioned modifiable and non‐modifiable factors is essential to develop culturally tailored public health interventions to improve CRC prevention and early detection among PRH.

### 4.4 Study Strengths, Limitations, and Implications for Future Research

The present study had notable strengths, including that the data on PRH obtained from the PRCCR, which is not included in SEER, was coded, gathered, and reported in accordance with the NAACCR guidelines, making it comparable to the data obtained from SEER. Additionally, to assess the diversity among Hispanic populations, our study clearly distinguished between Hispanic individuals living in the United States and those living in Puerto Rico. While we could not completely disaggregate the United States Hispanic population by country of origin, we provided critical insights into the roles that Hispanic subgroups may play in the overall EOCRC incidence trends by focusing on PRH. In addition to Puerto Ricans, PRH includes immigrant individuals primarily from the Dominican Republic (58.5%) and Cuba (11.2%). Other regions of origin include South America (14.6%), Central America (6.6%), and Europe (4.0%).

However, certain limitations must also be considered, including missing information on racial and ethnic identification, tumor location, histology subtype, and stage of diagnosis. This missing data may have limited our ability to explore molecular profiles and mechanisms contributing to the observed disparities. PRH had the highest percentage of cases with missing information on tumor location and stage at diagnosis, which could limit our analyses when considering these variables. Racial and ethnic classifications pose an intrinsic limitation as these are self‐reported based on social constructs, cultural backgrounds, and matching surnames (e.g., SEER). As people may identify with one or more races and ethnicities, “categories should not be considered absolute or viewed in isolation” [59]. One of the most important comparisons in CRC incidence trends made in this study was based on the stage at diagnosis. To be able to include all CRC cases from 2000 to 2021, we used data from SEER 17, as SEER 22 only provides stage‐specific data for cases diagnosed starting in 2004. While we acknowledge that SEER 22 covers a higher percentage of the United States population, SEER 17 covers a substantial percentage of population (approximately 26.5%) and was essential for our analysis as it offers a broader time range, ensuring that we could capture the complete trend in CRC incidence, including those diagnosed before 2004.

## 5 Conclusions

Hispanic is used as a blanket term to encompass persons of Cuban, Mexican, Puerto Rican, South or Central American, or other Spanish culture or origin, regardless of cultural and racial identities. Similarly, NHAI/AN is an umbrella term that includes individuals representing more than 600 federal‐ or state‐recognized tribes [60]. While both groups exhibit significant disparities in CRC incidence, this broad categorization underscores the importance of studying subpopulations individually, as unique genetic and environmental factors can lead to substantial differences in CRC incidence trends across heterogeneous subgroups.

In sum, we present updated CRC incidence data from PRH, and our findings underscore the need for research efforts that examine not only genetics and biology but also the environmental and behavioral factors that may drive the CRC incidence disparities observed. This study calls for a deeper reflection on the unique challenges particular populations face and how these may directly contribute to disparities in CRC incidence, particularly in EOCRC. Moving forward, more granular research is needed to consider factors like ancestral origin, in addition to other modifiable and unmodifiable risk factors, to guide the development of culturally tailored interventions aimed at addressing CRC disparities and improving cancer control.

## Author Contributions

Luis D. Borrero‐Garcia: project administration; investigation; writing – original draft; writing – review and editing. Marylyn Moró‐Carrión: investigation; validation; visualization; writing – review and editing. Carlos R. Torres‐Cintrón: conceptualization; data curation; formal analysis; methodology; visualization; writing – review and editing. Hilmaris Centeno‐Girona: validation: writing – review and editing. Victoria Perez: investigation; writing – original draft. Taymaraliz Santos‐Colón: investigation; visualization; writing – original draft. María González‐Pons: conceptualization; funding acquisition; investigation; methodology; supervision; validation; visualization; writing – original draft; writing – review and editing.

## Ethics Statement

This study was approved by the University of Puerto Rico Comprehensive Cancer Center Institutional Review Board (IRB # 2022‐10‐88).

## Consent

The authors have nothing to report.

## Conflicts of Interest

The authors declare no conflicts of interest.

## Supporting information


**Table S1.** Comparison of Trends in Incidence Using Parallelism and Coincident Tests. The comparison was performed pairwise between different race and ethnicity, sex, age group, and stage of diagnosis. *p* values ≤ 0.05 are significant. Coincident test results were not significant for any comparisons and are therefore not displayed.

## Data Availability

The data analyzed in this study are available upon request through the Puerto Rico Central Cancer Registry and the Surveillance, Epidemiology, and End Results (SEER) Program.
