# Correction to “Disparities in Colorectal Cancer Incidence Trends Among Hispanics Living in Puerto Rico (2000–2021): A Comparison With Surveillance, Epidemiology, and End Results (SEER) Database”

**DOI:** 10.1002/cam4.72122

**Published:** 2026-07-14

L. D. Borrero‐Garcia , M. Moró‐Carrión , C. R. Torres‐Cintrón , et al., “Disparities in Colorectal Cancer Incidence Trends Among Hispanics Living in Puerto Rico (2000–2021): A Comparison With Surveillance, Epidemiology, and End Results (SEER) Database,” Cancer Medicine 14, no. 8 (2025): e70851, 10.1002/cam4.70851 40249262 PMC12007180

In the Funding section, the award number for the funding source “Center for the Promotion of Cancer Health Equity (CePCHE, NIGMS award number 1P20GM148324)” was incorrect. This should have read: “Center for the Promotion of Cancer Health Equity (CePCHE, NIGMS award number P20GM148324)”.

We apologize for this error.

