# Supplementary material for: Disparities in Colorectal Cancer Incidence Trends Among Hispanics Living in Puerto Rico (2000–2021): A Comparison With Surveillance, Epidemiology, and End Results (SEER) Database
Source: Cancer Med. 2025 Apr 18;14(8):e70851. doi: 10.1002/cam4.70851 (PMC12007180; doi:10.1002/cam4.70851)
Supplement: Supplementary file 1 — Table S1. Comparison of Trends in Incidence Using Parallelism and Coincident Tests. The comparison was performed pairwise between different race and ethnicity, sex, age group, and stage of diagnosis. p values ≤ 0.05 are significant. Coincident test results were not significant for any comparisons and are therefore not displayed. [file CAM4-14-e70851-s001.docx]

**Supplementary Table 1. Comparison of Trends in Incidence Using Parallelism and Coincidence Tests.** The comparison was performed pairwise between different race and ethnicity, sex, age group, and stage of diagnosis. P-values ≤ 0.05 are significant. Coincidence test results were not significant for any comparisons and are therefore not displayed.

| Cohort 1 | Cohort 2 | P-Value |
| --- | --- | --- |
| NHW / " M" / 20-49 / Localized | NHW / " M" / 20-49 / Regional | **0.00022** |
| NHW / " M" / 20-49 / Localized | NHW / " M" / 20-49 / Distant | **0.00022** |
| NHW / " M" / 20-49 / Localized | NHW / " M" / 20-49 / Unstaged | **0.01133** |
| NHW / " M" / 20-49 / Localized | NHW / " M" / 20-49 / TOTAL | **0.00022** |
| NHW / " M" / 20-49 / Regional | NHW / " M" / 20-49 / Distant | **0.00044** |
| NHW / " M" / 20-49 / Regional | NHW / " M" / 20-49 / Unstaged | **0.00267** |
| NHW / " M" / 20-49 / Regional | NHW / " M" / 20-49 / TOTAL | 0.12089 |
| NHW / " M" / 20-49 / Distant | NHW / " M" / 20-49 / Unstaged | **0.00022** |
| NHW / " M" / 20-49 / Distant | NHW / " M" / 20-49 / TOTAL | **0.00022** |
| NHW / " M" / 20-49 / Unstaged | NHW / " M" / 20-49 / TOTAL | **0.002** |
| NHW / " M" / 50+ / Localized | NHW / " M" / 50+ / Regional | **0.00044** |
| NHW / " M" / 50+ / Localized | NHW / " M" / 50+ / Distant | **0.00022** |
| NHW / " M" / 50+ / Localized | NHW / " M" / 50+ / Unstaged | **0.00022** |
| NHW / " M" / 50+ / Localized | NHW / " M" / 50+ / TOTAL | **0.00022** |
| NHW / " M" / 50+ / Regional | NHW / " M" / 50+ / Distant | **0.00022** |
| NHW / " M" / 50+ / Regional | NHW / " M" / 50+ / Unstaged | **0.00044** |
| NHW / " M" / 50+ / Regional | NHW / " M" / 50+ / TOTAL | **0.01889** |
| NHW / " M" / 50+ / Distant | NHW / " M" / 50+ / Unstaged | **0.00067** |
| NHW / " M" / 50+ / Distant | NHW / " M" / 50+ / TOTAL | **0.00022** |
| NHW / " M" / 50+ / Unstaged | NHW / " M" / 50+ / TOTAL | **0.00044** |
| NHW / " M" / overall / Localized | NHW / " M" / overall / Regional | **0.00022** |
| NHW / " M" / overall / Localized | NHW / " M" / overall / Distant | **0.00022** |
| NHW / " M" / overall / Localized | NHW / " M" / overall / Unstaged | **0.00022** |
| NHW / " M" / overall / Localized | NHW / " M" / overall / TOTAL | **0.00022** |
| NHW / " M" / overall / Regional | NHW / " M" / overall / Distant | **0.00022** |
| NHW / " M" / overall / Regional | NHW / " M" / overall / Unstaged | **0.00133** |
| NHW / " M" / overall / Regional | NHW / " M" / overall / TOTAL | **0.00422** |
| NHW / " M" / overall / Distant | NHW / " M" / overall / Unstaged | **0.00089** |
| NHW / " M" / overall / Distant | NHW / " M" / overall / TOTAL | **0.00022** |
| NHW / " M" / overall / Unstaged | NHW / " M" / overall / TOTAL | **0.00022** |
| NHW / " F" / 20-49 / Localized | NHW / " F" / 20-49 / Regional | **0.00822** |
| NHW / " F" / 20-49 / Localized | NHW / " F" / 20-49 / Distant | **0.00022** |
| NHW / " F" / 20-49 / Localized | NHW / " F" / 20-49 / Unstaged | 0.18422 |
| NHW / " F" / 20-49 / Localized | NHW / " F" / 20-49 / TOTAL | **0.00111** |
| NHW / " F" / 20-49 / Regional | NHW / " F" / 20-49 / Distant | **0.00133** |
| NHW / " F" / 20-49 / Regional | NHW / " F" / 20-49 / Unstaged | 0.24222 |
| NHW / " F" / 20-49 / Regional | NHW / " F" / 20-49 / TOTAL | 0.69089 |
| NHW / " F" / 20-49 / Distant | NHW / " F" / 20-49 / Unstaged | 0.07911 |
| NHW / " F" / 20-49 / Distant | NHW / " F" / 20-49 / TOTAL | **0.00089** |
| NHW / " F" / 20-49 / Unstaged | NHW / " F" / 20-49 / TOTAL | 0.264 |
| NHW / " F" / 50+ / Localized | NHW / " F" / 50+ / Regional | 0.10978 |
| NHW / " F" / 50+ / Localized | NHW / " F" / 50+ / Distant | **0.00022** |
| NHW / " F" / 50+ / Localized | NHW / " F" / 50+ / Unstaged | **0.00067** |
| NHW / " F" / 50+ / Localized | NHW / " F" / 50+ / TOTAL | 0.10978 |
| NHW / " F" / 50+ / Regional | NHW / " F" / 50+ / Distant | **0.00044** |
| NHW / " F" / 50+ / Regional | NHW / " F" / 50+ / Unstaged | **0.00089** |
| NHW / " F" / 50+ / Regional | NHW / " F" / 50+ / TOTAL | 0.10956 |
| NHW / " F" / 50+ / Distant | NHW / " F" / 50+ / Unstaged | **0.00089** |
| NHW / " F" / 50+ / Distant | NHW / " F" / 50+ / TOTAL | **0.00022** |
| NHW / " F" / 50+ / Unstaged | NHW / " F" / 50+ / TOTAL | **0.00022** |
| NHW / " F" / overall / Localized | NHW / " F" / overall / Regional | **0.00267** |
| NHW / " F" / overall / Localized | NHW / " F" / overall / Distant | **0.00022** |
| NHW / " F" / overall / Localized | NHW / " F" / overall / Unstaged | **0.00022** |
| NHW / " F" / overall / Localized | NHW / " F" / overall / TOTAL | **0.00022** |
| NHW / " F" / overall / Regional | NHW / " F" / overall / Distant | **0.00022** |
| NHW / " F" / overall / Regional | NHW / " F" / overall / Unstaged | **0.00067** |
| NHW / " F" / overall / Regional | NHW / " F" / overall / TOTAL | 0.11289 |
| NHW / " F" / overall / Distant | NHW / " F" / overall / Unstaged | **0.00044** |
| NHW / " F" / overall / Distant | NHW / " F" / overall / TOTAL | **0.00022** |
| NHW / " F" / overall / Unstaged | NHW / " F" / overall / TOTAL | **0.00022** |
| NHW / M/F / 20-49 / Localized | NHW / M/F / 20-49 / Regional | **0.00022** |
| NHW / M/F / 20-49 / Localized | NHW / M/F / 20-49 / Distant | **0.00022** |
| NHW / M/F / 20-49 / Localized | NHW / M/F / 20-49 / Unstaged | **0.00133** |
| NHW / M/F / 20-49 / Localized | NHW / M/F / 20-49 / TOTAL | **0.00022** |
| NHW / M/F / 20-49 / Regional | NHW / M/F / 20-49 / Distant | **0.00022** |
| NHW / M/F / 20-49 / Regional | NHW / M/F / 20-49 / Unstaged | **0.00178** |
| NHW / M/F / 20-49 / Regional | NHW / M/F / 20-49 / TOTAL | 0.31622 |
| NHW / M/F / 20-49 / Distant | NHW / M/F / 20-49 / Unstaged | **0.00022** |
| NHW / M/F / 20-49 / Distant | NHW / M/F / 20-49 / TOTAL | **0.00022** |
| NHW / M/F / 20-49 / Unstaged | NHW / M/F / 20-49 / TOTAL | **0.00156** |
| NHW / M/F / 50+ / Localized | NHW / M/F / 50+ / Regional | **0.00711** |
| NHW / M/F / 50+ / Localized | NHW / M/F / 50+ / Distant | **0.00044** |
| NHW / M/F / 50+ / Localized | NHW / M/F / 50+ / Unstaged | **0.00022** |
| NHW / M/F / 50+ / Localized | NHW / M/F / 50+ / TOTAL | **0.00022** |
| NHW / M/F / 50+ / Regional | NHW / M/F / 50+ / Distant | **0.00022** |
| NHW / M/F / 50+ / Regional | NHW / M/F / 50+ / Unstaged | **0.00044** |
| NHW / M/F / 50+ / Regional | NHW / M/F / 50+ / TOTAL | 0.05444 |
| NHW / M/F / 50+ / Distant | NHW / M/F / 50+ / Unstaged | **0.00044** |
| NHW / M/F / 50+ / Distant | NHW / M/F / 50+ / TOTAL | **0.00022** |
| NHW / M/F / 50+ / Unstaged | NHW / M/F / 50+ / TOTAL | **0.00022** |
| NHW / M/F / overall / Localized | NHW / M/F / overall / Regional | **0.00444** |
| NHW / M/F / overall / Localized | NHW / M/F / overall / Distant | **0.00022** |
| NHW / M/F / overall / Localized | NHW / M/F / overall / Unstaged | **0.00022** |
| NHW / M/F / overall / Localized | NHW / M/F / overall / TOTAL | **0.00022** |
| NHW / M/F / overall / Regional | NHW / M/F / overall / Distant | **0.00022** |
| NHW / M/F / overall / Regional | NHW / M/F / overall / Unstaged | **0.00044** |
| NHW / M/F / overall / Regional | NHW / M/F / overall / TOTAL | **0.04578** |
| NHW / M/F / overall / Distant | NHW / M/F / overall / Unstaged | **0.00022** |
| NHW / M/F / overall / Distant | NHW / M/F / overall / TOTAL | **0.00022** |
| NHW / M/F / overall / Unstaged | NHW / M/F / overall / TOTAL | **0.00022** |
| NHB / " M" / 20-49 / Localized | NHB / " M" / 20-49 / Regional | 0.16311 |
| NHB / " M" / 20-49 / Localized | NHB / " M" / 20-49 / Distant | 0.07422 |
| NHB / " M" / 20-49 / Localized | NHB / " M" / 20-49 / TOTAL | 0.15311 |
| NHB / " M" / 20-49 / Regional | NHB / " M" / 20-49 / Distant | 0.568 |
| NHB / " M" / 20-49 / Regional | NHB / " M" / 20-49 / TOTAL | 0.35556 |
| NHB / " M" / 20-49 / Distant | NHB / " M" / 20-49 / TOTAL | **0.022** |
| NHB / " M" / 50+ / Localized | NHB / " M" / 50+ / Regional | **0.00022** |
| NHB / " M" / 50+ / Localized | NHB / " M" / 50+ / Distant | **0.00022** |
| NHB / " M" / 50+ / Localized | NHB / " M" / 50+ / Unstaged | **0.01267** |
| NHB / " M" / 50+ / Localized | NHB / " M" / 50+ / TOTAL | **0.02267** |
| NHB / " M" / 50+ / Regional | NHB / " M" / 50+ / Distant | **0.00022** |
| NHB / " M" / 50+ / Regional | NHB / " M" / 50+ / Unstaged | 0.46756 |
| NHB / " M" / 50+ / Regional | NHB / " M" / 50+ / TOTAL | **0.00067** |
| NHB / " M" / 50+ / Distant | NHB / " M" / 50+ / Unstaged | 0.09111 |
| NHB / " M" / 50+ / Distant | NHB / " M" / 50+ / TOTAL | **0.00022** |
| NHB / " M" / 50+ / Unstaged | NHB / " M" / 50+ / TOTAL | 0.31644 |
| NHB / " M" / overall / Localized | NHB / " M" / overall / Regional | **0.00044** |
| NHB / " M" / overall / Localized | NHB / " M" / overall / Distant | **0.00044** |
| NHB / " M" / overall / Localized | NHB / " M" / overall / Unstaged | **0.00467** |
| NHB / " M" / overall / Localized | NHB / " M" / overall / TOTAL | **0.022** |
| NHB / " M" / overall / Regional | NHB / " M" / overall / Distant | **0.00022** |
| NHB / " M" / overall / Regional | NHB / " M" / overall / Unstaged | 0.17733 |
| NHB / " M" / overall / Regional | NHB / " M" / overall / TOTAL | **0.00133** |
| NHB / " M" / overall / Distant | NHB / " M" / overall / Unstaged | **0.01933** |
| NHB / " M" / overall / Distant | NHB / " M" / overall / TOTAL | **0.00022** |
| NHB / " M" / overall / Unstaged | NHB / " M" / overall / TOTAL | 0.15844 |
| NHB / " F" / 20-49 / Localized | NHB / " F" / 20-49 / Regional | 0.46511 |
| NHB / " F" / 20-49 / Localized | NHB / " F" / 20-49 / Distant | 0.342 |
| NHB / " F" / 20-49 / Localized | NHB / " F" / 20-49 / Unstaged | 0.30689 |
| NHB / " F" / 20-49 / Localized | NHB / " F" / 20-49 / TOTAL | 0.97444 |
| NHB / " F" / 20-49 / Regional | NHB / " F" / 20-49 / Distant | 0.09622 |
| NHB / " F" / 20-49 / Regional | NHB / " F" / 20-49 / Unstaged | 0.43244 |
| NHB / " F" / 20-49 / Regional | NHB / " F" / 20-49 / TOTAL | 0.19111 |
| NHB / " F" / 20-49 / Distant | NHB / " F" / 20-49 / Unstaged | 0.20467 |
| NHB / " F" / 20-49 / Distant | NHB / " F" / 20-49 / TOTAL | 0.10911 |
| NHB / " F" / 20-49 / Unstaged | NHB / " F" / 20-49 / TOTAL | 0.29822 |
| NHB / " F" / 50+ / Localized | NHB / " F" / 50+ / Regional | **0.00311** |
| NHB / " F" / 50+ / Localized | NHB / " F" / 50+ / Distant | **0.00822** |
| NHB / " F" / 50+ / Localized | NHB / " F" / 50+ / Unstaged | **0.00622** |
| NHB / " F" / 50+ / Localized | NHB / " F" / 50+ / TOTAL | **0.046** |
| NHB / " F" / 50+ / Regional | NHB / " F" / 50+ / Distant | **0.00022** |
| NHB / " F" / 50+ / Regional | NHB / " F" / 50+ / Unstaged | **0.006** |
| NHB / " F" / 50+ / Regional | NHB / " F" / 50+ / TOTAL | **0.00689** |
| NHB / " F" / 50+ / Distant | NHB / " F" / 50+ / Unstaged | **0.00089** |
| NHB / " F" / 50+ / Distant | NHB / " F" / 50+ / TOTAL | **0.00022** |
| NHB / " F" / 50+ / Unstaged | NHB / " F" / 50+ / TOTAL | **0.00689** |
| NHB / " F" / overall / Localized | NHB / " F" / overall / Regional | **0.01044** |
| NHB / " F" / overall / Localized | NHB / " F" / overall / Distant | **0.01044** |
| NHB / " F" / overall / Localized | NHB / " F" / overall / Unstaged | **0.00378** |
| NHB / " F" / overall / Localized | NHB / " F" / overall / TOTAL | 0.07489 |
| NHB / " F" / overall / Regional | NHB / " F" / overall / Distant | **0.00022** |
| NHB / " F" / overall / Regional | NHB / " F" / overall / Unstaged | **0.00178** |
| NHB / " F" / overall / Regional | NHB / " F" / overall / TOTAL | 0.05822 |
| NHB / " F" / overall / Distant | NHB / " F" / overall / Unstaged | **0.00067** |
| NHB / " F" / overall / Distant | NHB / " F" / overall / TOTAL | **0.00022** |
| NHB / " F" / overall / Unstaged | NHB / " F" / overall / TOTAL | **0.00089** |
| NHB / M/F / 20-49 / Localized | NHB / M/F / 20-49 / Regional | 0.57133 |
| NHB / M/F / 20-49 / Localized | NHB / M/F / 20-49 / Distant | 0.06467 |
| NHB / M/F / 20-49 / Localized | NHB / M/F / 20-49 / Unstaged | 0.39467 |
| NHB / M/F / 20-49 / Localized | NHB / M/F / 20-49 / TOTAL | 0.29467 |
| NHB / M/F / 20-49 / Regional | NHB / M/F / 20-49 / Distant | 0.09956 |
| NHB / M/F / 20-49 / Regional | NHB / M/F / 20-49 / Unstaged | 0.26822 |
| NHB / M/F / 20-49 / Regional | NHB / M/F / 20-49 / TOTAL | 0.67667 |
| NHB / M/F / 20-49 / Distant | NHB / M/F / 20-49 / Unstaged | 0.12911 |
| NHB / M/F / 20-49 / Distant | NHB / M/F / 20-49 / TOTAL | **0.01978** |
| NHB / M/F / 20-49 / Unstaged | NHB / M/F / 20-49 / TOTAL | 0.25 |
| NHB / M/F / 50+ / Localized | NHB / M/F / 50+ / Regional | **0.00311** |
| NHB / M/F / 50+ / Localized | NHB / M/F / 50+ / Distant | **0.00089** |
| NHB / M/F / 50+ / Localized | NHB / M/F / 50+ / Unstaged | **0.00044** |
| NHB / M/F / 50+ / Localized | NHB / M/F / 50+ / TOTAL | **0.032** |
| NHB / M/F / 50+ / Regional | NHB / M/F / 50+ / Distant | **0.00022** |
| NHB / M/F / 50+ / Regional | NHB / M/F / 50+ / Unstaged | 0.11111 |
| NHB / M/F / 50+ / Regional | NHB / M/F / 50+ / TOTAL | **0.00089** |
| NHB / M/F / 50+ / Distant | NHB / M/F / 50+ / Unstaged | **0.00044** |
| NHB / M/F / 50+ / Distant | NHB / M/F / 50+ / TOTAL | **0.00022** |
| NHB / M/F / 50+ / Unstaged | NHB / M/F / 50+ / TOTAL | **0.01044** |
| NHB / M/F / overall / Localized | NHB / M/F / overall / Regional | **0.00089** |
| NHB / M/F / overall / Localized | NHB / M/F / overall / Distant | **0.00089** |
| NHB / M/F / overall / Localized | NHB / M/F / overall / Unstaged | **0.00111** |
| NHB / M/F / overall / Localized | NHB / M/F / overall / TOTAL | **0.04333** |
| NHB / M/F / overall / Regional | NHB / M/F / overall / Distant | **0.00022** |
| NHB / M/F / overall / Regional | NHB / M/F / overall / Unstaged | **0.00467** |
| NHB / M/F / overall / Regional | NHB / M/F / overall / TOTAL | **0.00756** |
| NHB / M/F / overall / Distant | NHB / M/F / overall / Unstaged | **0.00022** |
| NHB / M/F / overall / Distant | NHB / M/F / overall / TOTAL | **0.00022** |
| NHB / M/F / overall / Unstaged | NHB / M/F / overall / TOTAL | **0.00356** |
| NHAPI / " M" / 20-49 / Localized | NHAPI / " M" / 20-49 / Regional | 0.06644 |
| NHAPI / " M" / 20-49 / Localized | NHAPI / " M" / 20-49 / Distant | **0.00444** |
| NHAPI / " M" / 20-49 / Localized | NHAPI / " M" / 20-49 / TOTAL | **0.01289** |
| NHAPI / " M" / 20-49 / Regional | NHAPI / " M" / 20-49 / Distant | **0.02667** |
| NHAPI / " M" / 20-49 / Regional | NHAPI / " M" / 20-49 / TOTAL | 0.17511 |
| NHAPI / " M" / 20-49 / Distant | NHAPI / " M" / 20-49 / TOTAL | **0.032** |
| NHAPI / " M" / 50+ / Localized | NHAPI / " M" / 50+ / Regional | **0.00778** |
| NHAPI / " M" / 50+ / Localized | NHAPI / " M" / 50+ / Distant | **0.00289** |
| NHAPI / " M" / 50+ / Localized | NHAPI / " M" / 50+ / Unstaged | **0.00533** |
| NHAPI / " M" / 50+ / Localized | NHAPI / " M" / 50+ / TOTAL | **0.00356** |
| NHAPI / " M" / 50+ / Regional | NHAPI / " M" / 50+ / Distant | 0.06489 |
| NHAPI / " M" / 50+ / Regional | NHAPI / " M" / 50+ / Unstaged | **0.00067** |
| NHAPI / " M" / 50+ / Regional | NHAPI / " M" / 50+ / TOTAL | 0.05378 |
| NHAPI / " M" / 50+ / Distant | NHAPI / " M" / 50+ / Unstaged | 0.134 |
| NHAPI / " M" / 50+ / Distant | NHAPI / " M" / 50+ / TOTAL | 0.13978 |
| NHAPI / " M" / 50+ / Unstaged | NHAPI / " M" / 50+ / TOTAL | **0.00511** |
| NHAPI / " M" / overall / Localized | NHAPI / " M" / overall / Regional | **0.00578** |
| NHAPI / " M" / overall / Localized | NHAPI / " M" / overall / Distant | **0.00244** |
| NHAPI / " M" / overall / Localized | NHAPI / " M" / overall / Unstaged | **0.01089** |
| NHAPI / " M" / overall / Localized | NHAPI / " M" / overall / TOTAL | **0.00089** |
| NHAPI / " M" / overall / Regional | NHAPI / " M" / overall / Distant | **0.03422** |
| NHAPI / " M" / overall / Regional | NHAPI / " M" / overall / Unstaged | **0.00356** |
| NHAPI / " M" / overall / Regional | NHAPI / " M" / overall / TOTAL | 0.08822 |
| NHAPI / " M" / overall / Distant | NHAPI / " M" / overall / Unstaged | 0.15844 |
| NHAPI / " M" / overall / Distant | NHAPI / " M" / overall / TOTAL | 0.05822 |
| NHAPI / " M" / overall / Unstaged | NHAPI / " M" / overall / TOTAL | **0.01022** |
| NHAPI / " F" / 20-49 / Localized | NHAPI / " F" / 20-49 / Regional | 0.18978 |
| NHAPI / " F" / 20-49 / Localized | NHAPI / " F" / 20-49 / Distant | 0.05133 |
| NHAPI / " F" / 20-49 / Localized | NHAPI / " F" / 20-49 / TOTAL | 0.094 |
| NHAPI / " F" / 20-49 / Regional | NHAPI / " F" / 20-49 / Distant | 0.62311 |
| NHAPI / " F" / 20-49 / Regional | NHAPI / " F" / 20-49 / TOTAL | 0.21133 |
| NHAPI / " F" / 20-49 / Distant | NHAPI / " F" / 20-49 / TOTAL | 0.17333 |
| NHAPI / " F" / 50+ / Localized | NHAPI / " F" / 50+ / Regional | **0.00044** |
| NHAPI / " F" / 50+ / Localized | NHAPI / " F" / 50+ / Distant | **0.01556** |
| NHAPI / " F" / 50+ / Localized | NHAPI / " F" / 50+ / Unstaged | **0.00356** |
| NHAPI / " F" / 50+ / Localized | NHAPI / " F" / 50+ / TOTAL | **0.00067** |
| NHAPI / " F" / 50+ / Regional | NHAPI / " F" / 50+ / Distant | **0.04622** |
| NHAPI / " F" / 50+ / Regional | NHAPI / " F" / 50+ / Unstaged | **0.00022** |
| NHAPI / " F" / 50+ / Regional | NHAPI / " F" / 50+ / TOTAL | **0.00044** |
| NHAPI / " F" / 50+ / Distant | NHAPI / " F" / 50+ / Unstaged | **0.00044** |
| NHAPI / " F" / 50+ / Distant | NHAPI / " F" / 50+ / TOTAL | 0.15378 |
| NHAPI / " F" / 50+ / Unstaged | NHAPI / " F" / 50+ / TOTAL | **0.00022** |
| NHAPI / " F" / overall / Localized | NHAPI / " F" / overall / Regional | **0.00067** |
| NHAPI / " F" / overall / Localized | NHAPI / " F" / overall / Distant | **0.002** |
| NHAPI / " F" / overall / Localized | NHAPI / " F" / overall / Unstaged | **0.00244** |
| NHAPI / " F" / overall / Localized | NHAPI / " F" / overall / TOTAL | **0.00044** |
| NHAPI / " F" / overall / Regional | NHAPI / " F" / overall / Distant | **0.02978** |
| NHAPI / " F" / overall / Regional | NHAPI / " F" / overall / Unstaged | **0.00044** |
| NHAPI / " F" / overall / Regional | NHAPI / " F" / overall / TOTAL | **0.00067** |
| NHAPI / " F" / overall / Distant | NHAPI / " F" / overall / Unstaged | **0.00956** |
| NHAPI / " F" / overall / Distant | NHAPI / " F" / overall / TOTAL | **0.01022** |
| NHAPI / " F" / overall / Unstaged | NHAPI / " F" / overall / TOTAL | **0.00022** |
| NHAPI / M/F / 20-49 / Localized | NHAPI / M/F / 20-49 / Regional | 0.084 |
| NHAPI / M/F / 20-49 / Localized | NHAPI / M/F / 20-49 / Distant | **0.00533** |
| NHAPI / M/F / 20-49 / Localized | NHAPI / M/F / 20-49 / Unstaged | 0.13222 |
| NHAPI / M/F / 20-49 / Localized | NHAPI / M/F / 20-49 / TOTAL | **0.01422** |
| NHAPI / M/F / 20-49 / Regional | NHAPI / M/F / 20-49 / Distant | 0.294 |
| NHAPI / M/F / 20-49 / Regional | NHAPI / M/F / 20-49 / Unstaged | 0.83333 |
| NHAPI / M/F / 20-49 / Regional | NHAPI / M/F / 20-49 / TOTAL | 0.29378 |
| NHAPI / M/F / 20-49 / Distant | NHAPI / M/F / 20-49 / Unstaged | 0.95444 |
| NHAPI / M/F / 20-49 / Distant | NHAPI / M/F / 20-49 / TOTAL | **0.01511** |
| NHAPI / M/F / 20-49 / Unstaged | NHAPI / M/F / 20-49 / TOTAL | 0.61644 |
| NHAPI / M/F / 50+ / Localized | NHAPI / M/F / 50+ / Regional | **0.00111** |
| NHAPI / M/F / 50+ / Localized | NHAPI / M/F / 50+ / Distant | **0.00178** |
| NHAPI / M/F / 50+ / Localized | NHAPI / M/F / 50+ / Unstaged | **0.00156** |
| NHAPI / M/F / 50+ / Localized | NHAPI / M/F / 50+ / TOTAL | **0.00067** |
| NHAPI / M/F / 50+ / Regional | NHAPI / M/F / 50+ / Distant | **0.00711** |
| NHAPI / M/F / 50+ / Regional | NHAPI / M/F / 50+ / Unstaged | **0.00022** |
| NHAPI / M/F / 50+ / Regional | NHAPI / M/F / 50+ / TOTAL | **0.00044** |
| NHAPI / M/F / 50+ / Distant | NHAPI / M/F / 50+ / Unstaged | **0.00533** |
| NHAPI / M/F / 50+ / Distant | NHAPI / M/F / 50+ / TOTAL | 0.10956 |
| NHAPI / M/F / 50+ / Unstaged | NHAPI / M/F / 50+ / TOTAL | **0.00022** |
| NHAPI / M/F / overall / Localized | NHAPI / M/F / overall / Regional | **0.00111** |
| NHAPI / M/F / overall / Localized | NHAPI / M/F / overall / Distant | **0.00044** |
| NHAPI / M/F / overall / Localized | NHAPI / M/F / overall / Unstaged | **0.00267** |
| NHAPI / M/F / overall / Localized | NHAPI / M/F / overall / TOTAL | **0.00022** |
| NHAPI / M/F / overall / Regional | NHAPI / M/F / overall / Distant | **0.008** |
| NHAPI / M/F / overall / Regional | NHAPI / M/F / overall / Unstaged | **0.00044** |
| NHAPI / M/F / overall / Regional | NHAPI / M/F / overall / TOTAL | **0.00022** |
| NHAPI / M/F / overall / Distant | NHAPI / M/F / overall / Unstaged | **0.03111** |
| NHAPI / M/F / overall / Distant | NHAPI / M/F / overall / TOTAL | **0.00111** |
| NHAPI / M/F / overall / Unstaged | NHAPI / M/F / overall / TOTAL | **0.00022** |
| NHAIAN / " M" / 20-49 / Regional | NHAIAN / " M" / 20-49 / Distant | 0.71067 |
| NHAIAN / " M" / 20-49 / Regional | NHAIAN / " M" / 20-49 / TOTAL | 0.56867 |
| NHAIAN / " M" / 20-49 / Distant | NHAIAN / " M" / 20-49 / TOTAL | 0.47467 |
| NHAIAN / " M" / 50+ / Localized | NHAIAN / " M" / 50+ / Regional | 0.208 |
| NHAIAN / " M" / 50+ / Localized | NHAIAN / " M" / 50+ / Distant | 0.15511 |
| NHAIAN / " M" / 50+ / Localized | NHAIAN / " M" / 50+ / TOTAL | 0.21533 |
| NHAIAN / " M" / 50+ / Regional | NHAIAN / " M" / 50+ / Distant | 0.36444 |
| NHAIAN / " M" / 50+ / Regional | NHAIAN / " M" / 50+ / TOTAL | 0.14422 |
| NHAIAN / " M" / 50+ / Distant | NHAIAN / " M" / 50+ / TOTAL | 0.202 |
| NHAIAN / " M" / overall / Localized | NHAIAN / " M" / overall / Regional | 0.15622 |
| NHAIAN / " M" / overall / Localized | NHAIAN / " M" / overall / Distant | 0.16422 |
| NHAIAN / " M" / overall / Localized | NHAIAN / " M" / overall / TOTAL | 0.456 |
| NHAIAN / " M" / overall / Regional | NHAIAN / " M" / overall / Distant | 0.15356 |
| NHAIAN / " M" / overall / Regional | NHAIAN / " M" / overall / TOTAL | 0.07844 |
| NHAIAN / " M" / overall / Distant | NHAIAN / " M" / overall / TOTAL | 0.42356 |
| NHAIAN / " F" / 20-49 / Localized | NHAIAN / " F" / 20-49 / Regional | 0.12956 |
| NHAIAN / " F" / 20-49 / Localized | NHAIAN / " F" / 20-49 / Distant | 0.41178 |
| NHAIAN / " F" / 20-49 / Localized | NHAIAN / " F" / 20-49 / TOTAL | 0.482 |
| NHAIAN / " F" / 20-49 / Regional | NHAIAN / " F" / 20-49 / Distant | 0.12222 |
| NHAIAN / " F" / 20-49 / Regional | NHAIAN / " F" / 20-49 / TOTAL | 0.12267 |
| NHAIAN / " F" / 20-49 / Distant | NHAIAN / " F" / 20-49 / TOTAL | 0.19244 |
| NHAIAN / " F" / 50+ / Localized | NHAIAN / " F" / 50+ / Regional | 0.5 |
| NHAIAN / " F" / 50+ / Localized | NHAIAN / " F" / 50+ / Distant | 0.066 |
| NHAIAN / " F" / 50+ / Localized | NHAIAN / " F" / 50+ / TOTAL | 0.82556 |
| NHAIAN / " F" / 50+ / Regional | NHAIAN / " F" / 50+ / Distant | **0.02222** |
| NHAIAN / " F" / 50+ / Regional | NHAIAN / " F" / 50+ / TOTAL | 0.10667 |
| NHAIAN / " F" / 50+ / Distant | NHAIAN / " F" / 50+ / TOTAL | **0.02733** |
| NHAIAN / " F" / overall / Localized | NHAIAN / " F" / overall / Regional | 0.39556 |
| NHAIAN / " F" / overall / Localized | NHAIAN / " F" / overall / Distant | **0.01378** |
| NHAIAN / " F" / overall / Localized | NHAIAN / " F" / overall / TOTAL | 0.22556 |
| NHAIAN / " F" / overall / Regional | NHAIAN / " F" / overall / Distant | **0.00778** |
| NHAIAN / " F" / overall / Regional | NHAIAN / " F" / overall / TOTAL | **0.04867** |
| NHAIAN / " F" / overall / Distant | NHAIAN / " F" / overall / TOTAL | **0.00356** |
| NHAIAN / M/F / 20-49 / Localized | NHAIAN / M/F / 20-49 / Regional | 0.09644 |
| NHAIAN / M/F / 20-49 / Localized | NHAIAN / M/F / 20-49 / Distant | 0.64622 |
| NHAIAN / M/F / 20-49 / Localized | NHAIAN / M/F / 20-49 / TOTAL | 0.38311 |
| NHAIAN / M/F / 20-49 / Regional | NHAIAN / M/F / 20-49 / Distant | 0.56289 |
| NHAIAN / M/F / 20-49 / Regional | NHAIAN / M/F / 20-49 / TOTAL | 0.50022 |
| NHAIAN / M/F / 20-49 / Distant | NHAIAN / M/F / 20-49 / TOTAL | 0.76889 |
| NHAIAN / M/F / 50+ / Localized | NHAIAN / M/F / 50+ / Regional | 0.64756 |
| NHAIAN / M/F / 50+ / Localized | NHAIAN / M/F / 50+ / Distant | **0.01222** |
| NHAIAN / M/F / 50+ / Localized | NHAIAN / M/F / 50+ / Unstaged | **0.03533** |
| NHAIAN / M/F / 50+ / Localized | NHAIAN / M/F / 50+ / TOTAL | 0.48111 |
| NHAIAN / M/F / 50+ / Regional | NHAIAN / M/F / 50+ / Distant | **0.00244** |
| NHAIAN / M/F / 50+ / Regional | NHAIAN / M/F / 50+ / Unstaged | **0.01667** |
| NHAIAN / M/F / 50+ / Regional | NHAIAN / M/F / 50+ / TOTAL | 0.09489 |
| NHAIAN / M/F / 50+ / Distant | NHAIAN / M/F / 50+ / Unstaged | 0.17 |
| NHAIAN / M/F / 50+ / Distant | NHAIAN / M/F / 50+ / TOTAL | **0.00578** |
| NHAIAN / M/F / 50+ / Unstaged | NHAIAN / M/F / 50+ / TOTAL | **0.03556** |
| NHAIAN / M/F / overall / Localized | NHAIAN / M/F / overall / Regional | 0.13533 |
| NHAIAN / M/F / overall / Localized | NHAIAN / M/F / overall / Distant | **0.00333** |
| NHAIAN / M/F / overall / Localized | NHAIAN / M/F / overall / Unstaged | 0.05578 |
| NHAIAN / M/F / overall / Localized | NHAIAN / M/F / overall / TOTAL | 0.79333 |
| NHAIAN / M/F / overall / Regional | NHAIAN / M/F / overall / Distant | **0.00067** |
| NHAIAN / M/F / overall / Regional | NHAIAN / M/F / overall / Unstaged | 0.15733 |
| NHAIAN / M/F / overall / Regional | NHAIAN / M/F / overall / TOTAL | **0.03422** |
| NHAIAN / M/F / overall / Distant | NHAIAN / M/F / overall / Unstaged | 0.31044 |
| NHAIAN / M/F / overall / Distant | NHAIAN / M/F / overall / TOTAL | **0.00333** |
| NHAIAN / M/F / overall / Unstaged | NHAIAN / M/F / overall / TOTAL | 0.22667 |
| HIS / " M" / 20-49 / Localized | HIS / " M" / 20-49 / Regional | **0.04378** |
| HIS / " M" / 20-49 / Localized | HIS / " M" / 20-49 / Distant | **0.00044** |
| HIS / " M" / 20-49 / Localized | HIS / " M" / 20-49 / Unstaged | 0.09822 |
| HIS / " M" / 20-49 / Localized | HIS / " M" / 20-49 / TOTAL | **0.002** |
| HIS / " M" / 20-49 / Regional | HIS / " M" / 20-49 / Distant | **0.042** |
| HIS / " M" / 20-49 / Regional | HIS / " M" / 20-49 / Unstaged | 0.92911 |
| HIS / " M" / 20-49 / Regional | HIS / " M" / 20-49 / TOTAL | 0.83978 |
| HIS / " M" / 20-49 / Distant | HIS / " M" / 20-49 / Unstaged | 0.19356 |
| HIS / " M" / 20-49 / Distant | HIS / " M" / 20-49 / TOTAL | **0.00356** |
| HIS / " M" / 20-49 / Unstaged | HIS / " M" / 20-49 / TOTAL | 0.85533 |
| HIS / " M" / 50+ / Localized | HIS / " M" / 50+ / Regional | **0.006** |
| HIS / " M" / 50+ / Localized | HIS / " M" / 50+ / Distant | **0.00044** |
| HIS / " M" / 50+ / Localized | HIS / " M" / 50+ / Unstaged | **0.00156** |
| HIS / " M" / 50+ / Localized | HIS / " M" / 50+ / TOTAL | **0.00089** |
| HIS / " M" / 50+ / Regional | HIS / " M" / 50+ / Distant | **0.00756** |
| HIS / " M" / 50+ / Regional | HIS / " M" / 50+ / Unstaged | **0.04689** |
| HIS / " M" / 50+ / Regional | HIS / " M" / 50+ / TOTAL | 0.14022 |
| HIS / " M" / 50+ / Distant | HIS / " M" / 50+ / Unstaged | 0.06356 |
| HIS / " M" / 50+ / Distant | HIS / " M" / 50+ / TOTAL | **0.00022** |
| HIS / " M" / 50+ / Unstaged | HIS / " M" / 50+ / TOTAL | **0.04489** |
| HIS / " M" / overall / Localized | HIS / " M" / overall / Regional | **0.00267** |
| HIS / " M" / overall / Localized | HIS / " M" / overall / Distant | **0.00022** |
| HIS / " M" / overall / Localized | HIS / " M" / overall / Unstaged | **0.00067** |
| HIS / " M" / overall / Localized | HIS / " M" / overall / TOTAL | **0.00067** |
| HIS / " M" / overall / Regional | HIS / " M" / overall / Distant | **0.00311** |
| HIS / " M" / overall / Regional | HIS / " M" / overall / Unstaged | 0.07644 |
| HIS / " M" / overall / Regional | HIS / " M" / overall / TOTAL | 0.14244 |
| HIS / " M" / overall / Distant | HIS / " M" / overall / Unstaged | 0.07378 |
| HIS / " M" / overall / Distant | HIS / " M" / overall / TOTAL | **0.00022** |
| HIS / " M" / overall / Unstaged | HIS / " M" / overall / TOTAL | 0.05844 |
| HIS / " F" / 20-49 / Localized | HIS / " F" / 20-49 / Regional | 0.19244 |
| HIS / " F" / 20-49 / Localized | HIS / " F" / 20-49 / Distant | 0.07711 |
| HIS / " F" / 20-49 / Localized | HIS / " F" / 20-49 / Unstaged | 0.13511 |
| HIS / " F" / 20-49 / Localized | HIS / " F" / 20-49 / TOTAL | 0.08333 |
| HIS / " F" / 20-49 / Regional | HIS / " F" / 20-49 / Distant | 0.18956 |
| HIS / " F" / 20-49 / Regional | HIS / " F" / 20-49 / Unstaged | 0.23956 |
| HIS / " F" / 20-49 / Regional | HIS / " F" / 20-49 / TOTAL | 0.932 |
| HIS / " F" / 20-49 / Distant | HIS / " F" / 20-49 / Unstaged | 0.81756 |
| HIS / " F" / 20-49 / Distant | HIS / " F" / 20-49 / TOTAL | 0.17822 |
| HIS / " F" / 20-49 / Unstaged | HIS / " F" / 20-49 / TOTAL | 0.31511 |
| HIS / " F" / 50+ / Localized | HIS / " F" / 50+ / Regional | 0.65022 |
| HIS / " F" / 50+ / Localized | HIS / " F" / 50+ / Distant | **0.00978** |
| HIS / " F" / 50+ / Localized | HIS / " F" / 50+ / Unstaged | **0.04822** |
| HIS / " F" / 50+ / Localized | HIS / " F" / 50+ / TOTAL | 0.09511 |
| HIS / " F" / 50+ / Regional | HIS / " F" / 50+ / Distant | **0.02822** |
| HIS / " F" / 50+ / Regional | HIS / " F" / 50+ / Unstaged | 0.07711 |
| HIS / " F" / 50+ / Regional | HIS / " F" / 50+ / TOTAL | 0.55422 |
| HIS / " F" / 50+ / Distant | HIS / " F" / 50+ / Unstaged | 0.52044 |
| HIS / " F" / 50+ / Distant | HIS / " F" / 50+ / TOTAL | **0.016** |
| HIS / " F" / 50+ / Unstaged | HIS / " F" / 50+ / TOTAL | 0.08489 |
| HIS / " F" / overall / Localized | HIS / " F" / overall / Regional | 0.27244 |
| HIS / " F" / overall / Localized | HIS / " F" / overall / Distant | **0.00444** |
| HIS / " F" / overall / Localized | HIS / " F" / overall / Unstaged | 0.05644 |
| HIS / " F" / overall / Localized | HIS / " F" / overall / TOTAL | **0.02978** |
| HIS / " F" / overall / Regional | HIS / " F" / overall / Distant | **0.00911** |
| HIS / " F" / overall / Regional | HIS / " F" / overall / Unstaged | 0.14111 |
| HIS / " F" / overall / Regional | HIS / " F" / overall / TOTAL | 0.15133 |
| HIS / " F" / overall / Distant | HIS / " F" / overall / Unstaged | 0.69378 |
| HIS / " F" / overall / Distant | HIS / " F" / overall / TOTAL | **0.00444** |
| HIS / " F" / overall / Unstaged | HIS / " F" / overall / TOTAL | 0.14911 |
| HIS / M/F / 20-49 / Localized | HIS / M/F / 20-49 / Regional | 0.05911 |
| HIS / M/F / 20-49 / Localized | HIS / M/F / 20-49 / Distant | **0.00133** |
| HIS / M/F / 20-49 / Localized | HIS / M/F / 20-49 / Unstaged | **0.048** |
| HIS / M/F / 20-49 / Localized | HIS / M/F / 20-49 / TOTAL | **0.00778** |
| HIS / M/F / 20-49 / Regional | HIS / M/F / 20-49 / Distant | **0.04533** |
| HIS / M/F / 20-49 / Regional | HIS / M/F / 20-49 / Unstaged | 0.59556 |
| HIS / M/F / 20-49 / Regional | HIS / M/F / 20-49 / TOTAL | 0.63333 |
| HIS / M/F / 20-49 / Distant | HIS / M/F / 20-49 / Unstaged | 0.47556 |
| HIS / M/F / 20-49 / Distant | HIS / M/F / 20-49 / TOTAL | **0.00111** |
| HIS / M/F / 20-49 / Unstaged | HIS / M/F / 20-49 / TOTAL | 0.73867 |
| HIS / M/F / 50+ / Localized | HIS / M/F / 50+ / Regional | 0.11644 |
| HIS / M/F / 50+ / Localized | HIS / M/F / 50+ / Distant | **0.00067** |
| HIS / M/F / 50+ / Localized | HIS / M/F / 50+ / Unstaged | **0.00111** |
| HIS / M/F / 50+ / Localized | HIS / M/F / 50+ / TOTAL | **0.01978** |
| HIS / M/F / 50+ / Regional | HIS / M/F / 50+ / Distant | **0.002** |
| HIS / M/F / 50+ / Regional | HIS / M/F / 50+ / Unstaged | **0.012** |
| HIS / M/F / 50+ / Regional | HIS / M/F / 50+ / TOTAL | 0.30067 |
| HIS / M/F / 50+ / Distant | HIS / M/F / 50+ / Unstaged | **0.03511** |
| HIS / M/F / 50+ / Distant | HIS / M/F / 50+ / TOTAL | **0.00556** |
| HIS / M/F / 50+ / Unstaged | HIS / M/F / 50+ / TOTAL | **0.03511** |
| HIS / M/F / overall / Localized | HIS / M/F / overall / Regional | **0.01756** |
| HIS / M/F / overall / Localized | HIS / M/F / overall / Distant | **0.00022** |
| HIS / M/F / overall / Localized | HIS / M/F / overall / Unstaged | **0.00044** |
| HIS / M/F / overall / Localized | HIS / M/F / overall / TOTAL | **0.00489** |
| HIS / M/F / overall / Regional | HIS / M/F / overall / Distant | **0.00022** |
| HIS / M/F / overall / Regional | HIS / M/F / overall / Unstaged | **0.01578** |
| HIS / M/F / overall / Regional | HIS / M/F / overall / TOTAL | 0.31444 |
| HIS / M/F / overall / Distant | HIS / M/F / overall / Unstaged | **0.00889** |
| HIS / M/F / overall / Distant | HIS / M/F / overall / TOTAL | **0.00067** |
| HIS / M/F / overall / Unstaged | HIS / M/F / overall / TOTAL | **0.04267** |
| USA / " M" / 20-49 / Localized | USA / " M" / 20-49 / Regional | **0.00022** |
| USA / " M" / 20-49 / Localized | USA / " M" / 20-49 / Distant | **0.00022** |
| USA / " M" / 20-49 / Localized | USA / " M" / 20-49 / Unstaged | **0.00267** |
| USA / " M" / 20-49 / Localized | USA / " M" / 20-49 / TOTAL | **0.00022** |
| USA / " M" / 20-49 / Regional | USA / " M" / 20-49 / Distant | **0.00022** |
| USA / " M" / 20-49 / Regional | USA / " M" / 20-49 / Unstaged | **0.00333** |
| USA / " M" / 20-49 / Regional | USA / " M" / 20-49 / TOTAL | 0.11667 |
| USA / " M" / 20-49 / Distant | USA / " M" / 20-49 / Unstaged | **0.00022** |
| USA / " M" / 20-49 / Distant | USA / " M" / 20-49 / TOTAL | **0.00044** |
| USA / " M" / 20-49 / Unstaged | USA / " M" / 20-49 / TOTAL | **0.00222** |
| USA / " M" / 50+ / Localized | USA / " M" / 50+ / Regional | **0.00511** |
| USA / " M" / 50+ / Localized | USA / " M" / 50+ / Distant | **0.00022** |
| USA / " M" / 50+ / Localized | USA / " M" / 50+ / Unstaged | **0.00022** |
| USA / " M" / 50+ / Localized | USA / " M" / 50+ / TOTAL | **0.00022** |
| USA / " M" / 50+ / Regional | USA / " M" / 50+ / Distant | **0.00022** |
| USA / " M" / 50+ / Regional | USA / " M" / 50+ / Unstaged | **0.00022** |
| USA / " M" / 50+ / Regional | USA / " M" / 50+ / TOTAL | **0.01867** |
| USA / " M" / 50+ / Distant | USA / " M" / 50+ / Unstaged | **0.00044** |
| USA / " M" / 50+ / Distant | USA / " M" / 50+ / TOTAL | **0.00022** |
| USA / " M" / 50+ / Unstaged | USA / " M" / 50+ / TOTAL | **0.00022** |
| USA / " M" / overall / Localized | USA / " M" / overall / Regional | **0.00044** |
| USA / " M" / overall / Localized | USA / " M" / overall / Distant | **0.00022** |
| USA / " M" / overall / Localized | USA / " M" / overall / Unstaged | **0.00022** |
| USA / " M" / overall / Localized | USA / " M" / overall / TOTAL | **0.00067** |
| USA / " M" / overall / Regional | USA / " M" / overall / Distant | **0.00022** |
| USA / " M" / overall / Regional | USA / " M" / overall / Unstaged | **0.00022** |
| USA / " M" / overall / Regional | USA / " M" / overall / TOTAL | **0.01578** |
| USA / " M" / overall / Distant | USA / " M" / overall / Unstaged | **0.00044** |
| USA / " M" / overall / Distant | USA / " M" / overall / TOTAL | **0.00022** |
| USA / " M" / overall / Unstaged | USA / " M" / overall / TOTAL | **0.00022** |
| USA / " F" / 20-49 / Localized | USA / " F" / 20-49 / Regional | **0.01956** |
| USA / " F" / 20-49 / Localized | USA / " F" / 20-49 / Distant | **0.00022** |
| USA / " F" / 20-49 / Localized | USA / " F" / 20-49 / Unstaged | 0.12533 |
| USA / " F" / 20-49 / Localized | USA / " F" / 20-49 / TOTAL | **0.00956** |
| USA / " F" / 20-49 / Regional | USA / " F" / 20-49 / Distant | **0.00044** |
| USA / " F" / 20-49 / Regional | USA / " F" / 20-49 / Unstaged | 0.878 |
| USA / " F" / 20-49 / Regional | USA / " F" / 20-49 / TOTAL | 0.75489 |
| USA / " F" / 20-49 / Distant | USA / " F" / 20-49 / Unstaged | 0.26756 |
| USA / " F" / 20-49 / Distant | USA / " F" / 20-49 / TOTAL | **0.00067** |
| USA / " F" / 20-49 / Unstaged | USA / " F" / 20-49 / TOTAL | 0.85978 |
| USA / " F" / 50+ / Localized | USA / " F" / 50+ / Regional | **0.01067** |
| USA / " F" / 50+ / Localized | USA / " F" / 50+ / Distant | **0.00022** |
| USA / " F" / 50+ / Localized | USA / " F" / 50+ / Unstaged | **0.00022** |
| USA / " F" / 50+ / Localized | USA / " F" / 50+ / TOTAL | **0.02956** |
| USA / " F" / 50+ / Regional | USA / " F" / 50+ / Distant | **0.00111** |
| USA / " F" / 50+ / Regional | USA / " F" / 50+ / Unstaged | **0.00044** |
| USA / " F" / 50+ / Regional | USA / " F" / 50+ / TOTAL | **0.01622** |
| USA / " F" / 50+ / Distant | USA / " F" / 50+ / Unstaged | **0.00044** |
| USA / " F" / 50+ / Distant | USA / " F" / 50+ / TOTAL | **0.00022** |
| USA / " F" / 50+ / Unstaged | USA / " F" / 50+ / TOTAL | **0.00022** |
| USA / " F" / overall / Localized | USA / " F" / overall / Regional | **0.00244** |
| USA / " F" / overall / Localized | USA / " F" / overall / Distant | **0.00022** |
| USA / " F" / overall / Localized | USA / " F" / overall / Unstaged | **0.00022** |
| USA / " F" / overall / Localized | USA / " F" / overall / TOTAL | **0.01111** |
| USA / " F" / overall / Regional | USA / " F" / overall / Distant | **0.00022** |
| USA / " F" / overall / Regional | USA / " F" / overall / Unstaged | **0.00022** |
| USA / " F" / overall / Regional | USA / " F" / overall / TOTAL | **0.03089** |
| USA / " F" / overall / Distant | USA / " F" / overall / Unstaged | **0.00067** |
| USA / " F" / overall / Distant | USA / " F" / overall / TOTAL | **0.00022** |
| USA / " F" / overall / Unstaged | USA / " F" / overall / TOTAL | **0.00022** |
| USA / M/F / 20-49 / Localized | USA / M/F / 20-49 / Regional | **0.00044** |
| USA / M/F / 20-49 / Localized | USA / M/F / 20-49 / Distant | **0.00022** |
| USA / M/F / 20-49 / Localized | USA / M/F / 20-49 / Unstaged | **0.00378** |
| USA / M/F / 20-49 / Localized | USA / M/F / 20-49 / TOTAL | **0.00022** |
| USA / M/F / 20-49 / Regional | USA / M/F / 20-49 / Distant | **0.00022** |
| USA / M/F / 20-49 / Regional | USA / M/F / 20-49 / Unstaged | **0.036** |
| USA / M/F / 20-49 / Regional | USA / M/F / 20-49 / TOTAL | 0.53644 |
| USA / M/F / 20-49 / Distant | USA / M/F / 20-49 / Unstaged | **0.00222** |
| USA / M/F / 20-49 / Distant | USA / M/F / 20-49 / TOTAL | **0.00044** |
| USA / M/F / 20-49 / Unstaged | USA / M/F / 20-49 / TOTAL | **0.01133** |
| USA / M/F / 50+ / Localized | USA / M/F / 50+ / Regional | **0.008** |
| USA / M/F / 50+ / Localized | USA / M/F / 50+ / Distant | **0.00022** |
| USA / M/F / 50+ / Localized | USA / M/F / 50+ / Unstaged | **0.00022** |
| USA / M/F / 50+ / Localized | USA / M/F / 50+ / TOTAL | **0.00067** |
| USA / M/F / 50+ / Regional | USA / M/F / 50+ / Distant | **0.00044** |
| USA / M/F / 50+ / Regional | USA / M/F / 50+ / Unstaged | **0.00022** |
| USA / M/F / 50+ / Regional | USA / M/F / 50+ / TOTAL | **0.01156** |
| USA / M/F / 50+ / Distant | USA / M/F / 50+ / Unstaged | **0.00022** |
| USA / M/F / 50+ / Distant | USA / M/F / 50+ / TOTAL | **0.00022** |
| USA / M/F / 50+ / Unstaged | USA / M/F / 50+ / TOTAL | **0.00022** |
| USA / M/F / overall / Localized | USA / M/F / overall / Regional | **0.01044** |
| USA / M/F / overall / Localized | USA / M/F / overall / Distant | **0.00022** |
| USA / M/F / overall / Localized | USA / M/F / overall / Unstaged | **0.00022** |
| USA / M/F / overall / Localized | USA / M/F / overall / TOTAL | **0.00044** |
| USA / M/F / overall / Regional | USA / M/F / overall / Distant | **0.00022** |
| USA / M/F / overall / Regional | USA / M/F / overall / Unstaged | **0.00022** |
| USA / M/F / overall / Regional | USA / M/F / overall / TOTAL | **0.01889** |
| USA / M/F / overall / Distant | USA / M/F / overall / Unstaged | **0.00044** |
| USA / M/F / overall / Distant | USA / M/F / overall / TOTAL | **0.00022** |
| USA / M/F / overall / Unstaged | USA / M/F / overall / TOTAL | **0.00022** |
| PR / " M" / 20-49 / Localized | PR / " M" / 20-49 / Regional | 0.09178 |
| PR / " M" / 20-49 / Localized | PR / " M" / 20-49 / Distant | 0.76978 |
| PR / " M" / 20-49 / Localized | PR / " M" / 20-49 / TOTAL | 0.11733 |
| PR / " M" / 20-49 / Regional | PR / " M" / 20-49 / Distant | **0.00067** |
| PR / " M" / 20-49 / Regional | PR / " M" / 20-49 / TOTAL | 0.074 |
| PR / " M" / 20-49 / Distant | PR / " M" / 20-49 / TOTAL | **0.01333** |
| PR / " M" / 50+ / Localized | PR / " M" / 50+ / Regional | **0.00022** |
| PR / " M" / 50+ / Localized | PR / " M" / 50+ / Distant | **0.00556** |
| PR / " M" / 50+ / Localized | PR / " M" / 50+ / Unstaged | 0.08067 |
| PR / " M" / 50+ / Localized | PR / " M" / 50+ / TOTAL | **0.00044** |
| PR / " M" / 50+ / Regional | PR / " M" / 50+ / Distant | **0.00133** |
| PR / " M" / 50+ / Regional | PR / " M" / 50+ / Unstaged | **0.00511** |
| PR / " M" / 50+ / Regional | PR / " M" / 50+ / TOTAL | **0.00489** |
| PR / " M" / 50+ / Distant | PR / " M" / 50+ / Unstaged | **0.00933** |
| PR / " M" / 50+ / Distant | PR / " M" / 50+ / TOTAL | **0.004** |
| PR / " M" / 50+ / Unstaged | PR / " M" / 50+ / TOTAL | 0.08956 |
| PR / " M" / overall / Localized | PR / " M" / overall / Regional | **0.00133** |
| PR / " M" / overall / Localized | PR / " M" / overall / Distant | **0.00178** |
| PR / " M" / overall / Localized | PR / " M" / overall / Unstaged | 0.08867 |
| PR / " M" / overall / Localized | PR / " M" / overall / TOTAL | **0.00022** |
| PR / " M" / overall / Regional | PR / " M" / overall / Distant | **0.00044** |
| PR / " M" / overall / Regional | PR / " M" / overall / Unstaged | **0.006** |
| PR / " M" / overall / Regional | PR / " M" / overall / TOTAL | **0.01111** |
| PR / " M" / overall / Distant | PR / " M" / overall / Unstaged | **0.00578** |
| PR / " M" / overall / Distant | PR / " M" / overall / TOTAL | **0.00067** |
| PR / " M" / overall / Unstaged | PR / " M" / overall / TOTAL | 0.11978 |
| PR / " F" / 20-49 / Localized | PR / " F" / 20-49 / Regional | 0.34733 |
| PR / " F" / 20-49 / Localized | PR / " F" / 20-49 / Distant | 0.07489 |
| PR / " F" / 20-49 / Localized | PR / " F" / 20-49 / TOTAL | **0.026** |
| PR / " F" / 20-49 / Regional | PR / " F" / 20-49 / Distant | 0.12733 |
| PR / " F" / 20-49 / Regional | PR / " F" / 20-49 / TOTAL | 0.89822 |
| PR / " F" / 20-49 / Distant | PR / " F" / 20-49 / TOTAL | **0.01222** |
| PR / " F" / 50+ / Localized | PR / " F" / 50+ / Regional | **0.00533** |
| PR / " F" / 50+ / Localized | PR / " F" / 50+ / Distant | **0.00044** |
| PR / " F" / 50+ / Localized | PR / " F" / 50+ / Unstaged | 0.06022 |
| PR / " F" / 50+ / Localized | PR / " F" / 50+ / TOTAL | **0.012** |
| PR / " F" / 50+ / Regional | PR / " F" / 50+ / Distant | **0.00089** |
| PR / " F" / 50+ / Regional | PR / " F" / 50+ / Unstaged | 0.16622 |
| PR / " F" / 50+ / Regional | PR / " F" / 50+ / TOTAL | **0.00267** |
| PR / " F" / 50+ / Distant | PR / " F" / 50+ / Unstaged | **0.02222** |
| PR / " F" / 50+ / Distant | PR / " F" / 50+ / TOTAL | **0.00022** |
| PR / " F" / 50+ / Unstaged | PR / " F" / 50+ / TOTAL | 0.054 |
| PR / " F" / overall / Localized | PR / " F" / overall / Regional | **0.00556** |
| PR / " F" / overall / Localized | PR / " F" / overall / Distant | **0.00022** |
| PR / " F" / overall / Localized | PR / " F" / overall / Unstaged | 0.07267 |
| PR / " F" / overall / Localized | PR / " F" / overall / TOTAL | **0.00489** |
| PR / " F" / overall / Regional | PR / " F" / overall / Distant | **0.00044** |
| PR / " F" / overall / Regional | PR / " F" / overall / Unstaged | **0.03511** |
| PR / " F" / overall / Regional | PR / " F" / overall / TOTAL | 0.05844 |
| PR / " F" / overall / Distant | PR / " F" / overall / Unstaged | **0.01711** |
| PR / " F" / overall / Distant | PR / " F" / overall / TOTAL | **0.00022** |
| PR / " F" / overall / Unstaged | PR / " F" / overall / TOTAL | **0.04067** |
| PR / M/F / 20-49 / Localized | PR / M/F / 20-49 / Regional | 0.14156 |
| PR / M/F / 20-49 / Localized | PR / M/F / 20-49 / Distant | 0.30044 |
| PR / M/F / 20-49 / Localized | PR / M/F / 20-49 / TOTAL | 0.20533 |
| PR / M/F / 20-49 / Regional | PR / M/F / 20-49 / Distant | **0.00489** |
| PR / M/F / 20-49 / Regional | PR / M/F / 20-49 / TOTAL | 0.31244 |
| PR / M/F / 20-49 / Distant | PR / M/F / 20-49 / TOTAL | **0.00289** |
| PR / M/F / 50+ / Localized | PR / M/F / 50+ / Regional | **0.00244** |
| PR / M/F / 50+ / Localized | PR / M/F / 50+ / Distant | **0.00089** |
| PR / M/F / 50+ / Localized | PR / M/F / 50+ / Unstaged | 0.08333 |
| PR / M/F / 50+ / Localized | PR / M/F / 50+ / TOTAL | **0.00111** |
| PR / M/F / 50+ / Regional | PR / M/F / 50+ / Distant | **0.00022** |
| PR / M/F / 50+ / Regional | PR / M/F / 50+ / Unstaged | **0.01022** |
| PR / M/F / 50+ / Regional | PR / M/F / 50+ / TOTAL | **0.00778** |
| PR / M/F / 50+ / Distant | PR / M/F / 50+ / Unstaged | **0.00733** |
| PR / M/F / 50+ / Distant | PR / M/F / 50+ / TOTAL | **0.00022** |
| PR / M/F / 50+ / Unstaged | PR / M/F / 50+ / TOTAL | 0.13711 |
| PR / M/F / overall / Localized | PR / M/F / overall / Regional | **0.00467** |
| PR / M/F / overall / Localized | PR / M/F / overall / Distant | **0.00044** |
| PR / M/F / overall / Localized | PR / M/F / overall / Unstaged | 0.09333 |
| PR / M/F / overall / Localized | PR / M/F / overall / TOTAL | **0.002** |
| PR / M/F / overall / Regional | PR / M/F / overall / Distant | **0.00022** |
| PR / M/F / overall / Regional | PR / M/F / overall / Unstaged | **0.00956** |
| PR / M/F / overall / Regional | PR / M/F / overall / TOTAL | **0.01733** |
| PR / M/F / overall / Distant | PR / M/F / overall / Unstaged | **0.00689** |
| PR / M/F / overall / Distant | PR / M/F / overall / TOTAL | **0.00022** |
